# Electrically tunable giant Nernst effect in two-dimensional van der Waals heterostructures

**DOI:** 10.1038/s41565-024-01717-y

**Published:** 2024-07-02

**Authors:** Gabriele Pasquale, Zhe Sun, Guilherme Migliato Marega, Kenji Watanabe, Takashi Taniguchi, Andras Kis

**Affiliations:** 1 Institute of Electrical and Microengineering, École Polytechnique Fédérale de Lausanne (EPFL), Lausanne, Switzerland; 2 Institute of Materials Science and Engineering, École Polytechnique Fédérale de Lausanne (EPFL), Lausanne, Switzerland; 3 Research Center for Electronic and Optical Materials, National Institute for Materials Science, Tsukuba, Japan; 4 Research Center for Materials Nanoarchitectonics, National Institute for Materials Science, Tsukuba, Japan

**Keywords:** Two-dimensional materials, Materials for devices

## Abstract

The Nernst effect, a transverse thermoelectric phenomenon, has attracted significant attention for its potential in energy conversion, thermoelectrics and spintronics. However, achieving high performance and versatility at low temperatures remains elusive. Here we demonstrate a large and electrically tunable Nernst effect by combining the electrical properties of graphene with the semiconducting characteristics of indium selenide in a field-effect geometry. Our results establish a new platform for exploring and manipulating this thermoelectric effect, showcasing the first electrical tunability with an on/off ratio of 10^3^. Moreover, photovoltage measurements reveal a stronger photo-Nernst signal in the graphene/indium selenide heterostructure compared with individual components. Remarkably, we observe a record-high Nernst coefficient of 66.4 μV K^−1^ T^−1^ at ultralow temperatures and low magnetic fields, an important step towards applications in quantum information and low-temperature emergent phenomena.

## Abstract (web summary)

A highly tunable Nernst effect has been demonstrated in graphene/indium selenide devices, achieving a record Nernst coefficient at ultralow temperatures, highlighting its potential for quantum technologies and low-temperature applications.

## Main

The investigation of thermoelectricity traces its origins back to the mid-nineteenth century when Lord Kelvin embarked on a quest to comprehend it as a quasi-thermodynamic phenomenon. A notable milestone in this journey occurred in 1931 with the formulation of reciprocal relations by Onsager^1^. Such relations established crucial connections, including the Kelvin relation between Seebeck and Peltier coefficients and the Bridgman relation linking the Nernst and Ettingshausen effects^2^. Practical applications, however, have been limited to date. Nevertheless, recent technological advancements and promising applications in energy conversion, thermoelectrics and spintronics have renewed interest in thermoelectric phenomena^3–7^. One such effect is the Nernst–Ettingshausen effect, which manifests itself as a transverse electric field, known as the Nernst voltage, generated by the Lorentz force acting on charge carriers in the presence of a temperature gradient and a magnetic field. Among the recently investigated materials, topological semimetals show promise for efficient thermoelectric cooling via the Nernst–Ettingshausen effect^3,8^. Such materials are characterized by zero or slight band overlap, and high carrier mobilities that are beneficial for enhancing thermoelectric effects. On the other hand, difficulties in measuring the transverse thermoelectric effects have slowed down the progress compared with its longitudinal counterpart^9–12^, despite extensive work on reaching high thermoelectric figures of merit^3,5,6^.

Achieving a sizable and tunable Nernst effect at ultralow temperatures remains an ongoing challenge^13,14^, especially in the sub-Kelvin regime^10^, where it is gaining increasing momentum due to potential applications in quantum technologies. Within the context of qubit circuits where precise thermal control is paramount^15,16^, the ability to convert localized heat sources, both internal and external to the circuit, into controllable electric signals emerges as a pivotal asset^15,17^. Hence, this capability has the potential to contribute to the fine-tuning of quantum systems, although the full extent of its impact and the complexities involved in manipulating quantum states remain subjects of ongoing research.

To date, implementing such low-temperature thermal management methods remains challenging due to significant magnetic-field constraints, thereby requiring advancements in materials and techniques. In fact, conventional materials exhibit limited Nernst response at ultralow temperatures^12,18^, motivating the search for novel materials with large and tunable Nernst effect in such conditions^18^.

Here we show a large and tunable Nernst effect by combining graphene with the metal monochalcogenide indium selenide (InSe) assembled in a field-effect geometry. After the observation of the photoinduced Nernst effect in graphene^19^, further studies have provided insights into the nature of the effect and its applications^20^. Among several candidates, InSe is chosen due to a combination of desirable properties, such as high electron mobility^21^, low resistivity and its peculiar band topology, which is predicted to give rise to enhanced thermoelectric phenomena^22–26^. By taking advantage of the exceptional electrical conductivity of graphene and the intriguing semiconducting properties of InSe (refs. ^27–30^), we demonstrate the first electrically tunable Nernst effect with an on/off ratio of 10^3^ in a field-effect structure. This signal arises when the sample is subject to a magnetic field and a temperature gradient generated by laser illumination. Furthermore, by measuring the signal in a different geometry, using graphene as electrodes and InSe as a channel material, we obtain a Nernst coefficient of 66.4 μV K^−1^ T^−1^, which represents the highest value observed at ultralow temperatures and low magnetic fields.

## Device structure and Nernst effect in Gr/InSe heterostructure

**Fig. 1:**
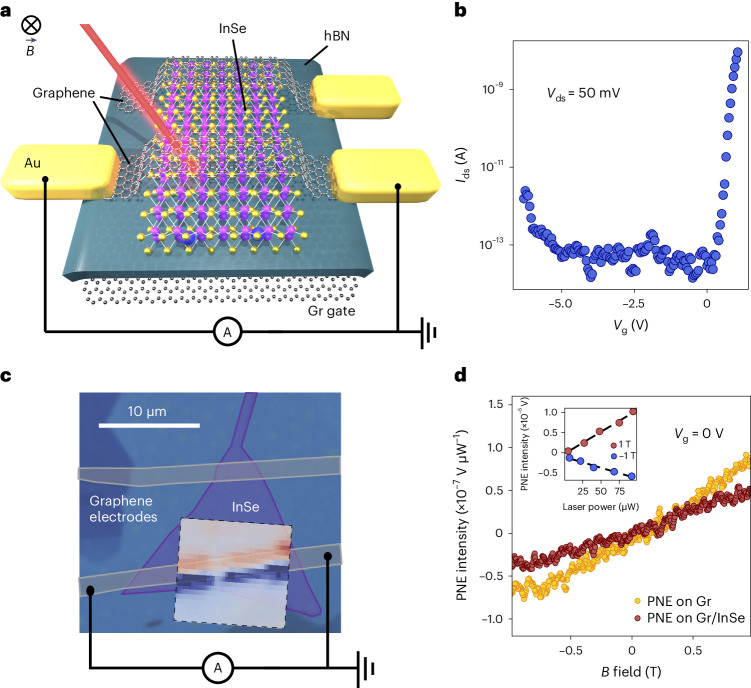
Device schematics and basic characterization. **a**, Device schematic representing a fully encapsulated few-layer InSe channel, with graphene electrodes. One of the electrodes is contacted on both sides with gold contacts to perform measurements of graphene, affected by InSe. The scanning photocurrent maps are performed at 100 mK, with 50 µW laser power and an out-of-plane magnetic field of 1 T, unless specified otherwise. **b**, Electron transport characteristics of a typical few-layer InSe device, in this case 3L InSe, performed at a low temperature under 50 mV of bias. **c**, Optical micrograph of a 3L InSe device with graphene electrodes. An examplary measurement of the photo-Nernst effect at 1 T measured on graphene is shown overlapped with the region of interest. **d**, Linear magnetic-field dependence of the Nernst effect when the illumination is on graphene alone (dark yellow) and Gr/InSe (dark red). The inset shows the power dependence of the Nernst effect on Gr and sign change for an opposite magnetic field, confirming the origin of the effect as the photoinduced Nernst effect (PNE).

To ensure the high quality and reliability of our devices, we employ a fabrication process that involves encapsulating γ-phase InSe flakes within hexagonal boron nitride (hBN) and using graphene or few-layer graphite (FLG) flakes as electrodes^31,32^. The device architecture is designed to incorporate a graphene electrode spanning the entire length of the InSe flake, enabling the measurements of graphene properties and their response to the proximity of InSe (ref. ^33^). Additionally, an FLG bottom gate is utilized to modulate the carrier density in the semiconductor. The schematic of the Gr/InSe heterostructure is illustrated in Fig. 1a.

We present the longitudinal transport characteristics of a representative device (Fig. 1b), demonstrating clear ambipolar transport in few-layer InSe (ref. ^30^). We perform thermoelectric power measurements at 100 mK in a dilution refrigerator by performing scanning photocurrent (and photovoltage) measurements, in both d.c. and a.c. using a lock-in amplifier (Methods and Supplementary Note 6). Figure 1c depicts a false-colour image of a three-layer Gr/InSe device, with a superimposed spatial map illustrating the photoinduced Nernst effect signal within the field of view. The Nernst effect is characterized by the emergence of a transverse current (voltage) on laser illumination at the edges of the channel^19^. Such illumination induces an uncompensated thermal gradient that drives the current along the channel when the sample is subjected to a magnetic field^19,20^. Importantly, this signal changes sign at opposite magnetic-field polarities and remains consistent across the length of the channel as the laser is scanned, exhibiting a uniform profile that changes sign at opposite edges of the flake, in accordance with the principles outlined in the Shockley–Ramo theorem^34^.

The photoinduced Nernst effect current can be described by the equation ${I}_{{{\rm{ph}}}}=\beta {NB}{\rho }_{{xx}}^{-1}\times \Delta {T}_{{{\rm{av}}}}$, where *N* represents the Nernst coefficient, *B* is the applied magnetic field perpendicular to the channel, ${\rho }_{{xx}}^{-1}$ is the inverse of the longitudinal resistivity and Δ*T*_av_ is the average temperature difference induced across the edges by means of laser illumination, whereas *β* < 1 accounts for the geometric factor that incorporates contact resistance^19^. Throughout our work, we conduct the measurements of both Nernst effect current and voltage, with a detailed derivation and formalism presented in Supplementary Note 3. To be able to compare the signal across multiple devices and conditions, we define the photoinduced Nernst effect intensity as the photovoltage or photocurrent signal normalized over the magnetic field and laser power. The magnetic field and laser powers are kept at 1 T and 50 µW, respectively, unless specified otherwise. To confirm the nature of the effect, we demonstrate the linear and antisymmetric behaviours of the Nernst effect with respect to the applied magnetic field, as well as the linearity of the effect by increasing the laser power^19^ (Fig. 1d). Additional characterization and measurements can be found in Supplementary Note 3. In particular, the Nernst effect can be measured both on bare graphene and within the heterostructure region, emphasizing that the presence of InSe does not quench the signal. Importantly, all the measurements are conducted with a laser wavelength of *λ* = 532 nm and under a magnetic field of 1 T, unless stated otherwise. At such laser powers, the background temperature of the sample stabilizes at around 100 mK. In our discussion, we exclude the effect of excitons in InSe as a possible source of the observed effect in graphene since no bias voltage is applied to dissociate electron–hole pairs and the signal is absent at zero magnetic field.

**Fig. 2:**
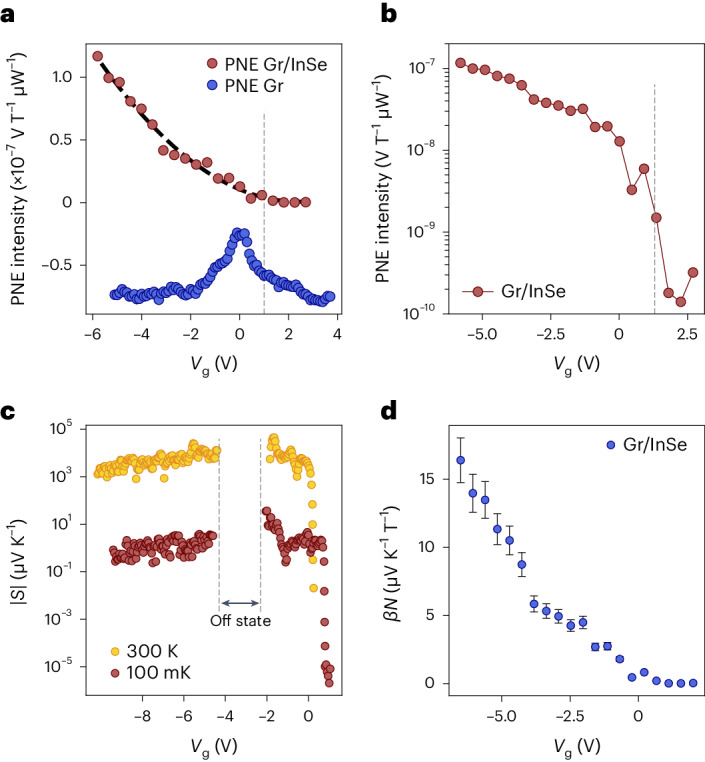
Photo-Nernst effect and thermoelectric performance. **a**, Nernst effect intensity normalized by laser power measured on the 3L InSe/Gr heterostructure (dark red) as a function of gate voltage. The Nernst effect of bare graphene is shown as a comparison (blue), and is offset for clarity. **b**, Nernst effect intensity of the heterostructure plotted in the logarithmic scale to highlight the sizable on/off ratio. The effect can be switched on and off by changing the carrier density within our device. The grey dashed lines represent the onset of n-type conduction, analogous to **a**. **c**, Seebeck coefficient calculated through the Mott relations (Supplementary Note 10) as a function of gate voltage, shown for 300 K (yellow) and 100 mK (red). The vertical grey dashed lines represent the off state of the device, where the Seebeck coefficient cannot be defined due to the high resistance of the device, which becomes comparable with the input impedance of the instrument^38^. **d**, Gate-tunable Nernst coefficient in the Gr/InSe heterostructure. The plot represents a lower bound for the real value, due to *β*. The error bar reflects the error present in the determination of temperature (Supplementary Note 3).

To evaluate the Nernst response of the heterostructure, we compare the Nernst effect measured when shining laser light on bare graphene, with that obtained from the Gr/InSe heterostructure as a function of the gate voltage (Fig. 2a). The graphene signal aligns with previously reported findings^19,20^, exhibiting a peak feature at the Dirac point and decaying branches for both voltage polarities. In contrast, the signal within the Gr/InSe structure undergoes a dramatic change when varying the gate voltage from positive to negative values. The signal shown in Fig. 2 is recorded within the same set of measurements and within a constrained amount of time to limit spurious effects. To understand this result, we perform an in-depth analysis of the effect on the heterostructure. We record the Nernst effect signal as a function of the magnetic field and gate voltage, yielding the expected linear and antisymmetric behaviours as measured previously for bare graphene (Fig. 1d). However, the slope of this effect shows significant changes on the modulation of gate voltage. In particular, at positive gate voltages where InSe exhibits high conductivity, the Nernst effect is strongly suppressed. Conversely, at increasingly negative gate voltages, the slope increased well beyond the value observed at *V*_g_ = 0 V. By plotting the Nernst effect intensity at 1 T of the Gr/InSe heterostructure as a function of the gate voltage on a logarithmic scale, a clear modulation of the effect that can be switched on and off is revealed, with a ratio of ~10^3^ (Fig. 2b). The on/off ratio of the gate modulation of the Nernst effect intensity is defined as the ratio between the maximum and minimum values of the signal, similar to the standards of field-effect transistor devices^35,36^. Since no quenching of the effect is observed on bare graphene, as previously investigated^19,20^, we attribute this effect to the presence of the InSe flake, as further elaborated below.

We can compute the Seebeck coefficient of InSe using the traditional Mott relation^37^: ${S}_{{\rm{InSe}}}=\,\frac{{\uppi }^{2}{k}_{{\rm{b}}}^{2}T}{3e}\frac{1}{G}\frac{{{\rm{d}}G}}{{\rm{d}}{V}_{{\rm{g}}}}\frac{{\rm{d}}{V}_{{\rm{g}}}}{{\rm{d}}{E}_{{\rm{F}}}}\,$, where *k*_b_ is the Boltzmann constant, *T* is the temperature, *e* is the electron charge, *G* is the conductivity, *V*_g_ is the gate voltage and *E*_F_ is the Fermi energy. The factor $\frac{{\rm{d}}{V}_{{\rm{g}}}}{{\rm{d}}{E}_{{\rm{F}}}}$ was calculated based on our previous work^29^ (Supplementary Note 5 provides detailed information). We acknowledge that although the Mott formula may not yield results in perfect agreement with the experimental ones as reported previously^24^, the observed discrepancies typically remain within a factor of 2. Importantly, these deviations do not alter the core findings and conclusions of our work, namely, an electrically tunable Nernst device operating at low magnetic fields and millikelvin temperatures (Supplementary Note 3).

By keeping into account the gate dependence of the Seebeck effect, we can derive the values for the Seebeck coefficient of InSe, which are shown for room temperature (yellow) and for 100 mK (red) in Fig. 2c (Supplementary Note 3). The room-temperature values of the Seebeck coefficient of few-layer InSe are comparable with the best values reported for monolayer semiconducting materials^38,39^. Further, they exhibit a higher tunability ranging from a maximum value of –3.7 × 10^5^ µV K^–1^ to a minimum value of –4.1 × 10^–2^ µV K^–1^. Thus, a difference of seven orders of magnitude is present, compared with three orders of magnitude, as shown, for example, for monolayer MoS_2_ (ref. ^39^). We note that our devices are in the few-layer-thickness regime (3–5L), and we anticipate that devices with fewer layers could exhibit even more pronounced effects^26^. At low temperatures, the tunability remains significant, although the values are noticeably reduced compared with room temperature.

The high Seebeck coefficient of few-layer InSe relative to graphene gives rise to a substantial thermoelectric voltage at the interface on laser illumination that acts as an additional electric bias, resulting from the thermoelectric signal denoted by *V*_th_ = Δ*T* × (*S*_InSe_ – S_Gr_) (refs. ^39,40^). Hence, we can qualitatively elucidate the quenching of the Nernst effect signal at the interface for positive gate voltages, as the Seebeck coefficient of InSe becomes greatly reduced and comparable with that of graphene, leading to the suppression of the thermoelectric voltage caused by the disparity in Seebeck coefficients^41^. Conversely, the highly resistive state of InSe within the bandgap and its hole conduction state facilitates a substantial Seebeck coefficient, thereby preserving the thermal gradient at the interface. Furthermore, the presence of defects and impurities in the InSe layer can also alter the electronic properties of graphene. In such a process, if the charge carriers photogenerated in the graphene layer scatter off the defects and impurities in the InSe layer, it would effectively increase the local temperature when the Fermi level lies below the energy of the defect states in InSe (Supplementary Note 2).

From the Nernst effect equations (Supplementary Equations (9) and (10)), we can derive the lower bound of the Nernst coefficient for InSe. The values of *βN* obtained for the 3L InSe/Gr heterostructure are presented in Fig. 2d as a function of gate voltage. The Nernst coefficient can be effectively tuned by manipulating the carrier density, reaching a maximum value of 17.5 µV K^–1^ at negative voltages and becoming negligible at positive gate voltages. The error bar in the plot represents the effect of the error of the thermal gradient propagated to the Nernst coefficient. Remarkably, this wide range of tunability for the Nernst coefficient can enhance the Nernst effect of graphene to compete with the best materials available at comparable temperatures, namely, Bi and Bi–Sb alloys^14,42^. Hence, it holds great promise for applications of the Nernst effect in miniaturized devices, enabling the active switching of thermopower efficiency through an applied electric field.

## Giant Nernst response in field-effect geometry

**Fig. 3:**
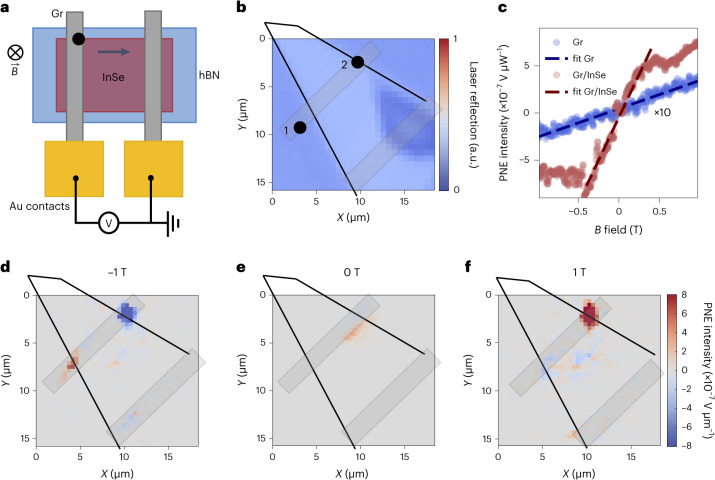
Scanning photovoltage map of the Nernst effect across an InSe channel. **a**, Device schematic showing the illumination of the Gr/5L-InSe heterostructure and electrical detection across the InSe channel. **b**, Laser reflectance map of the region of interest measured simultaneously with the scanning photovoltage map. This measurement allows us to correlate the position of the laser with the observed signal. The positions chosen to record the Nernst effect signal on graphene and on the Gr/InSe heterostructure are labelled as positions 1 and 2, respectively. **c**, Nernst effect signal recorded by varying the magnetic field and under 50 µW of laser illumination and *V*_g_ = 0 V in positions 1 and 2, shining light on the graphene electrode and on the heterostructure, respectively. The bare graphene signal is shown in blue, multiplied by 10 to better highlight the difference in slope between the two curves. The measurements are performed without any applied bias as it would obscure the Nernst effect, inducing other photovoltage mechanisms in the picture. **d**, Scanning photovoltage map showing the measured photovoltage across the full device at an applied out-of-plane electric field of –1 T. Here the temperature gradient is along the direction of the graphene electrode, orthogonal to the magnetic field and the measured potential. **e**,**f**, Scanning photovoltage maps shown for 0 T (**e**) and 1 T (**f**). Rectangles in **b**,**d**–**f** represent graphene contacts.

During our investigation, we noted the emergence of a Nernst signal when employing an alternative measurement geometry (Fig. 3a). This configuration involves the utilization of two graphene flakes as electrodes positioned on the InSe semiconducting channel, forming a field-effect structure. The system is illuminated with laser light, and a laser reflectance map of the device is shown in Fig. 3b, where the two positions labelled on the map represent the measurement sites on bare graphene (1) and the graphene/InSe heterostructure region (2). To quantify the effect in this geometry, we compare the signals obtained by illuminating the graphene alone (site 1) and the heterostructure (site 2) and varying the magnetic field (Fig. 3c). In particular, the signal recorded on the graphene electrode is comparable with that of the proximitized graphene discussed earlier (Fig. 2a). On the other hand, the signal recorded on the heterostructure shows a clear deviation from linearity when the magnetic field goes above ±0.5 T. To compare the effects, we restrict ourselves to the linear region, and we compute the slope of the photovoltage. Such a slope serves as a parameter for assessing the efficiency of the two effects, with a higher slope indicating a stronger effect. Remarkably, when the laser is directed onto the heterostructure, a significantly larger signal is observed with respect to the first site, and the ratio of the two slopes is approximately 42. The result is reproducible over several locations on the sample. This suggests that overall, the InSe channel facilitates a more efficient effect, yielding a calculated lower bound of the Nernst coefficient of 66.4 µV K^–1^ T^–1^ at 1 T.

To gain further insight, we record the scanning photovoltage map in the absence of bias and gate voltage. In particular, when the magnetic field is applied, we observe a strong signal arising on one of the graphene electrodes at the edges of InSe, as depicted in the scanning photocurrent maps for –1, 0 and 1 T magnetic fields (Fig. 3d–f, respectively). Here, the temperature gradient is along the direction of the graphene electrode, orthogonal to the channel. The observed signal arises on the application of a magnetic field, and both trend and sign of the measured photovoltage follow the Nernst effect geometry. One possible origin of the effect can be attributed to the known favourable interplay between the low Fermi energy of the system, a lower thermal conductivity with respect to graphene^24,25,43,44^ and its high electron mobility^6,27,28^. However, the precise mechanism behind this phenomenon requires further investigation. To gain a comprehensive understanding of the microscopic origins of this enhancement, rigorous theoretical investigations are also encouraged.

## Benchmarking of thermoelectric properties

**Fig. 4:**
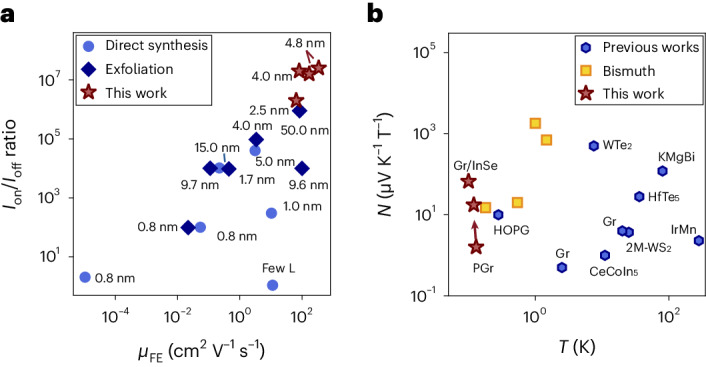
Thermoelectric benchmarking. **a**, Benchmarking of the field-effect two-terminal electron mobility and on/off ratio of InSe-based devices as recently reported in refs. ^28,45,51–57^ and refs. ^58,59^, respectively. Each point is labelled by the thickness of the InSe channel. The values measured in this work refer to the lateral electron transport and are indicated with red stars, showing a substantial improvement in both on/off ratio and field-effect mobility. **b**, Low-temperature values of the Nernst coefficient for different materials^5,11,12,14,18,41,60–62^ and geometries^63,64^. In particular, bismuth is shown as yellow squares, since it possesses the best performance reported to date, to the best of our knowledge. The values measured in this work are shown as red stars. The red arrow indicates the tunability of the Nernst coefficient measured on graphene in proximity with InSe when changing the gate voltage, outperforming the bismuth counterpart. All of the values taken from the literature are displayed at 1 T, to have a meaningful comparison, since most of these materials possess high Nernst values for different ranges of magnetic field.

One crucial parameter that allows for the observation of the Nernst-like effect in the geometry shown in Fig. 3a is the high electron mobility of our InSe channel. To evaluate the performance of our field-effect devices compared with the existing literature, we extract the room-temperature field-effect mobility and current on/off ratio for our InSe-based devices (Fig. 4a). The values considered for this comparison refer to the InSe thicknesses within the few-layer limit since the air sensitivity, inverted band curvature and effect of Van Hove singularity become relevant in this range of thicknesses. Our devices exhibit superior performance in both current on/off ratio and field-effect mobility compared with previous reports. In particular, we achieve a record on/off ratio of approximately 10^7^ and maximum field-effect mobility of approximately 150 cm^2^ V^–1^ s^–1^, surpassing the best values reported so far of approximately 10^5^ and 10 cm^2^ V^–1^ s^–1^, respectively (Fig. 4a)^45^. This performance enhancement results from improved InSe material quality (HQ Graphene; Methods) and device fabrication techniques. The high electron mobility in our devices enables the observation of the photoinduced Nernst effect for the first time through a layered semiconductor channel, facilitated by the low resistivity of the few-layer InSe, which is comparable with that of graphene without an applied gate voltage^46^.

Moreover, we plot the Nernst coefficients in our devices and we compare them with the existing values present in the literature at their respective temperature (Fig. 4b). Our results show a wide electrical tunability of the Nernst effect across a wide range of values, reaching some of the best device performances reported so far. Moreover, the value of the Nernst coefficient obtained in the heterostructure region when measuring in the geometry shown in Fig. 3a as detailed above represents, to the best of our knowledge, the highest value observed for modest magnetic fields at temperatures below 200 mK. Thus, it establishes a new benchmark for the lowest operational temperature ever utilized successfully in Nernst measurements.

## Conclusion

In summary, our study presents the demonstration of a micrometre-sized thermoelectric device harnessing the photoinduced Nernst effect, displaying exceptional performance even at ultralow temperatures of 100 mK, which were previously unattainable in this context. By harnessing the unique properties of the Gr/InSe heterostructure, we achieve a Nernst coefficient comparable with bismuth at ~200 mK and 1 T, and benefiting from the added advantage of tunability through carrier concentration modulation. Thus, leveraging the two-dimensional nature of our device, we achieve the first low-temperature, high-performance, tunable thermoelectric Nernst effect in a field-effect device. We note that the main advantage of having electrical tunability relies in the control and versatility it offers in practical applications. Since it can be achieved through standard electronic components, electrical control is straightforward to integrate into electronic devices and systems, making it compatible with existing technology.

In the traditional field-effect geometry (Fig. 3a), we observe an unprecedented record-high Nernst coefficient of 66.4 μV K^−1^ T^−1^ at 100 mK and 1 T, which is comparable with the Nernst coefficient values of materials currently employed at room temperature in commercial devices^47^. These findings not only establish Gr/InSe heterostructures as promising candidates for ultralow temperature operation and the investigation of emergent physics but also emphasize the significance of precise control and conversion of heat into electrical signals in such systems. In particular, the ability to convert localized heat sources into controllable electric signals could be implemented in qubit circuits as a thermal management technique, which is an active area of research efforts^15,17^.

By effectively bridging the gap between fundamental research and practical applications, our work provides a solid foundation for transformative advancements in quantum technologies, emergent phenomena and thermoelectric engineering^48–50^.

## Methods

### Device fabrication

The heterostructures utilized in this study were fabricated using the conventional dry transfer method. Initially, hBN and graphene/FLG (NGS) building blocks were obtained by mechanical exfoliation and deposition onto silicon oxide. Subsequently, all the components were assembled by starting from the uppermost hBN layer, which was lifted using a polycarbonate membrane on polydimethylsiloxane and carefully placed on top of an FLG bottom gate. The few-layer InSe (HQ Graphene) flakes were exfoliated onto polydimethylsiloxane (Gel-Pak) and distinguished based on their optical contrast. To prevent material degradation and contamination, all of these procedures were carried out within an argon-filled glovebox (inert). Once the sample was fully encapsulated, it underwent annealing at 340 °C in high vacuum with a pressure of 10^–6^ mbar for a duration of 6 h. Lastly, electrical contacts were fabricated by employing electron-beam lithography and depositing metal (Ti/Au) through evaporation with thickness of 2/100 nm.

### Optical and electrical measurements

All the measurements shown in this work were carried out under vacuum at 100 mK, unless specified otherwise. Scanning photocurrent and laser reflectance measurements were performed by focusing a laser on a spot of about 1 µm diameter on the sample. Multiple laser sources have been used for this purpose, and consistent results have been obtained for all the sources: a narrow-linewidth tunable continuous-wave laser (MSquared) and continuous-wave laser diodes (Thorlabs) with wavelengths centred at 780, 904 and 648 nm, for Nernst measurements, photocurrent measurements and thermal signal generation. In particular, for all the measurements reported in the main text, a laser wavelength of 532 nm of a continuous-wave laser is used. The incident power was varied from 1 to 300 µW for power dependence measurements and kept at 50 µW for the Nernst effect measurements shown in the main text, unless otherwise specified. All the data shown in the text are measured at 50 µW of laser power, unless stated otherwise, to allow a stable background temperature and clear comparison between the different samples. Transport measurements were carried out at room temperature and 80 mK with a Keithley 2636 sourcemeter. The 80 mK temperature was achieved inside a dilution fridge from Oxford Instruments, with a custom-made window and mirrors that allow us to perform optical and optoelectronic measurements. The background temperature is affected by the laser power, with a stable temperature of 100 mK when employing 50 µW. The electrical signal was detected by both d.c. and a.c. photocurrents (and photovoltage) with a Stanford Research SR830 lock-in amplifier, driven by a frequency of 727 Hz obtained using a synchronized laser chopper.

## Online content

Any methods, additional references, Nature Portfolio reporting summaries, source data, extended data, supplementary information, acknowledgements, peer review information; details of author contributions and competing interests; and statements of data and code availability are available at 10.1038/s41565-024-01717-y.

### Supplementary information

Supplementary Information Supplementary Notes 1–11, Figs. 1–19, Tables 1–3, Equations (1)–(14), text and references.

## Data Availability

The data that support the findings of this study are available from the corresponding author on reasonable request.
