# Supplementary material for: Electrically tunable giant Nernst effect in two-dimensional van der Waals heterostructures
Source: Nat Nanotechnol. 2024 Jul 2;19(7):941–7. doi: 10.1038/s41565-024-01717-y (PMC11286520; doi:10.1038/s41565-024-01717-y)
Supplement: Supplementary file 1 — Supplementary Notes 1–11, Figs. 1–19, Tables 1–3, Equations (1)–(14), text and references. [file 41565_2024_1717_MOESM1_ESM.pdf]

nature nanotechnology

Supplementary information https://doi.org/10.1038/s41565-024-01717-y

Electrically tunable giant Nernst effect in two-dimensional van der Waals heterostructures

In the format provided by the authors and unedited

Supplementary Information

for

Electrically tunable giant Nernst effect in two-dimensional van der Waals heterostructures

Table of contents

1. Device fabrication and characterization... 2
2. Photo-Nernst effect formalism and characterization.. 3
3. Thermoelectric signal: Seebeck and Nernst effect calculations... 7
4. Seebeck and Nernst Power factor... 10
5. Relation between Fermi energy change and carrier density in InSe.................................. 12
6. Experimental Setup.. 12
7. High-Field PNE.. 13
8. Complementary measurements.. 14
9. Possible origin of the enhancement and influence of the van Hove singularity………….. 16
10. Ultra-low temperature applications……………………………………………………..... 17
11. Simulation of heat transport in InSe/Graphene Heterostructure………………………… 18
12. References 22

1. Device fabrication and characterization

In this section, we provide an overview of the materials, the manufacturing process, and the analysis of the structural properties of the devices investigated in this work. Initially, we isolate all the constituent components on SiO_2, with the exception of the InSe flake, which is isolated on PDMS. Figure S1 illustrates the optical images of the flakes after exfoliation. After carefully selecting the flakes under the optical microscope, we proceed to assemble the devices using a conventional dry-stacking technique. Figure S1f shows the resultant device. The same fabrication procedure is employed for all the devices examined in this research.

In particular, graphene or few-layer graphite electrodes have been used. Our exfoliation method yields several as-exfoliated high aspect-ratio graphene flakes that are ideal to be employed directly as electrodes, as shown in Figure S1d. Such a step is crucial since it allows us to fabricate a full device without employing any kind of etching, air, or polymer exposure.

Typical hBN flakes and corresponding AFM height profiles are shown in Figure S2. The hBN flakes are selected to be within the 18 nm and 30 nm thickness range. A representative AFM morphology image is shown for the 3L InSe/Gr device in Figure S2c.

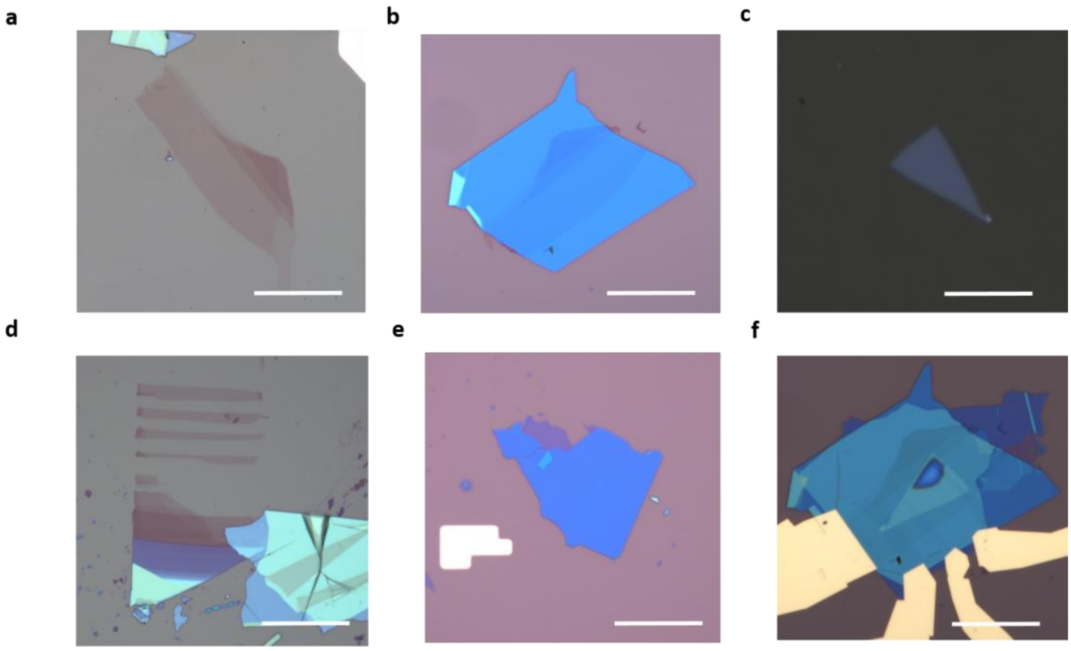

Figure S1. As exfoliated building blocks for the 5L device. a, Bottom few-layer graphite gate **b,** Bottom hBN flake transferred on the bottom gate, the thickness of 26 nm. **c,** Optical image of 5L InSe, thickness 4.1 nm. **d,** Graphene/few-layer graphite electrodes **e** Top hBN flake used to pick up the electrodes and close the heterostructure. **f,** Few-layer InSe device, completed with Ti/Au contacts. The scale bars are 20 µm.

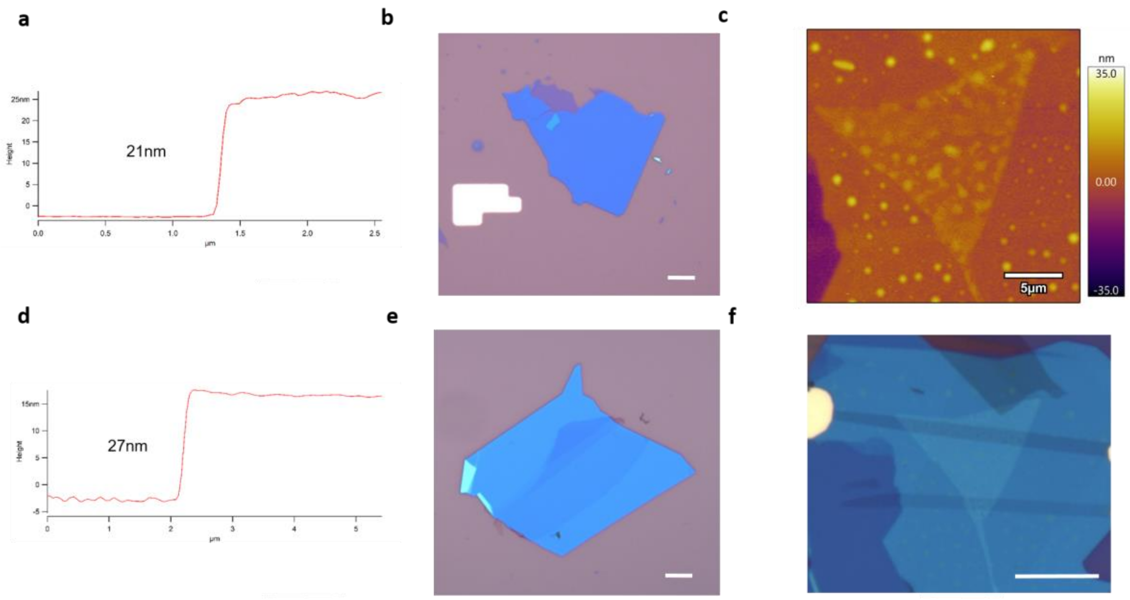

Figure S2. Device fabrication. (a-d) Thickness measurement and corresponding optical images (b-e) of two exemplary hBN flakes. (c) AFM image of the 3L Gr/InSe heterostructure. (f) Optical micrograph of the same device scanned in (c). The scale bars are 10 μm.

A typical few-layer InSe device shows very pronounced n-type conduction, even for very small bias voltages as shown in the main text in Figure 1. The overall resistivity values of n-type InSe are comparable to those of graphene as shown in Figure S3. Even closer values were reported for 5L and 6L InSe[1] where the resistivity is measured in the 4-wire geometry, thus yielding a more precise value that can remove the contact resistance contribution.

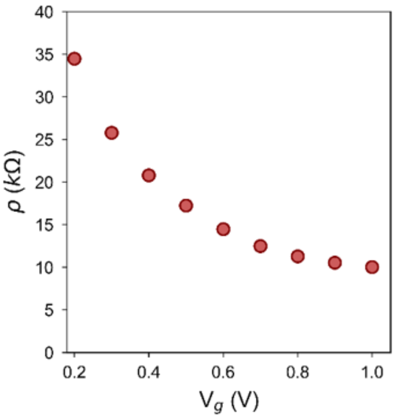

Figure S3. Electrical conductivity of InSe. The electrical conductivity of 3L InSe is plotted at different gate voltages. The bias voltage is kept to 20mV and the temperature is at 80K to compare with graphene.

2. Photo-Nernst effect formalism and characterization

The operation of photovoltaic cells is based on the spatial separation of photogenerated electrons and holes generated by an electric field and their diffusion to the contacts. However, in gapless materials like graphene, the full electron distribution rapidly thermalizes, eliminating the distinction between electrons and holes. Nevertheless, graphene devices can still produce photocurrent when light is focused on inhomogeneous regions or junctions. The photocurrent generated in graphene devices has been shown to have primarily a thermoelectric nature. The heating of electrons by the laser can be enhanced by slow energy transfer to the lattice due to the large optical phonon energy and high electron velocity.

In graphene, unlike in semiconductors, the transport of majority carriers to the contacts cannot be solely attributed to diffusion. Instead, in materials without a bandgap, a localized source of current density (referred to as j_{loc}) generates a global photocurrent (referred to as I_{ph}) by establishing an electric field that drives carriers from the surrounding region towards the contacts. This principle was discussed by Song and Levitov, who derived an equation for I_{ph} as an integral over j_{loc}, akin to the Shockley-Ramo theorem that explains the current generation between two conducting plates in the presence of charge movement.

Experimental investigations on graphene devices subjected to a magnetic field have verified the existence of the photo-Nernst effect, which leads to the generation of a photocurrent as postulated by this theorem[2]. When a magnetic field (B) perpendicular to the graphene plane is applied, a transverse current proportional to B tends to circulate around the region illuminated by the laser, perpendicular to the electron temperature gradient induced by the laser. The magnitude of the photocurrent remains consistent regardless of the distance from the contacts when the laser is positioned near an unbounded edge.

The observed variations in the photocurrent with respect to gate voltage and magnetic field align with conventional thermoelectric measurements conducted on graphene Hall bars, thus confirming the photo-Nernst nature of the observed response.

As was discussed by Cao et al.[2], for the case of a rectangular strip of isotropic 2D material, with electrodes at x = 0 and L and free edges at y = 0 and W, far from contacts one can compute the photocurrent as:

$$I_{ph} = -\frac{\beta}{L}\int\int\left[\left(\alpha_{xy} + r\alpha_{xx}\right)\frac{\partial T}{\partial y} + \left(\alpha_{xx} + r\alpha_{xy}\right)\frac{\partial T}{\partial x}\right]dx\,dy \tag{S1}$$

where $r = (\rho_{xy}/\rho_{xx})$, and α_{ij} are the components of the 2D thermoelectric tensor $\overleftrightarrow{\alpha}$, and the coefficient $\beta < 1$ depends on contact details.

The expression containing $\frac{\partial T}{\partial x}$ which possesses symmetry with respect to B yields a zero value upon integration when the temperature increase at the contacts is insignificant (or when it is identical at both ends). Conversely, the second term exhibits an asymmetry with respect to B and is directly proportional to the average temperature disparity ΔT_{av} between the two free boundaries, hence:

$$-\beta\left(\alpha_{xy} + r\alpha_{xx}\right)\frac{1}{L}\int_0^L dx\int_0^W \frac{\partial T}{\partial y}\,dy = -\,\beta\left(\alpha_{xy} + r\alpha_{xx}\right)\Delta T_{av} \tag{S2}$$

By considering the definition of the Nernst coefficient N as the ratio of the transverse electric field and the longitudinal temperature gradient multiplied by the magnetic field in the absence of charge current, we can deduce that $-\left(\alpha_{xy} + r\alpha_{xx}\right) = NB/\rho_{xx}$ for an isotropic material. Consequently, in the case of a rectangular device, when the laser is focused at a sufficient distance from the contacts, the following equations hold:

$$I_{ph} = \beta NB\rho_{xx}^{-1}\Delta T_{av} \tag{S3}$$

$$V_{ph} = \beta N\frac{l}{w}B\Delta T_{av} \tag{S4}$$

Where l and w are the length and width of the channel, respectively. Equations S3 and S4 describe the photocurrent (photovoltage) generated by the photo-Nernst effect. The usage of

one with respect to the other is dependent on the measurement schematic, and although formally equivalent, a subtle difference is present, as elucidated below.

First, for a photocurrent signal to be measured, a charge flow between the electrodes needs to exist, whereas a difference in potential can be present even in the absence of charge flow.

This means that the photovoltage will be more sensitive to the photogenerated potential, whereas the photocurrent is more strongly bound to the conduction channel. In particular, Equation S3 directly depends on the resistivity of the channel, while Equation S4 depends only on geometrical factors, thus providing a more sensitive tool. However, care must be taken in the photovoltage measurements since a signal can be observed even without good electrical contact with the channel. Thus, to verify that our electrodes are in good contact with the channel we perform transport measurements as a test for each pair of electrodes.

For the abovementioned reasons, we prioritized the photovoltage measurements in our work. On the other hand, when evaluating the effect of InSe as a channel, the current needs to flow directly within the semiconductor. We performed photocurrent measurements for this case to ensure such a charge flow in the InSe, and the result is shown in Figure S4.

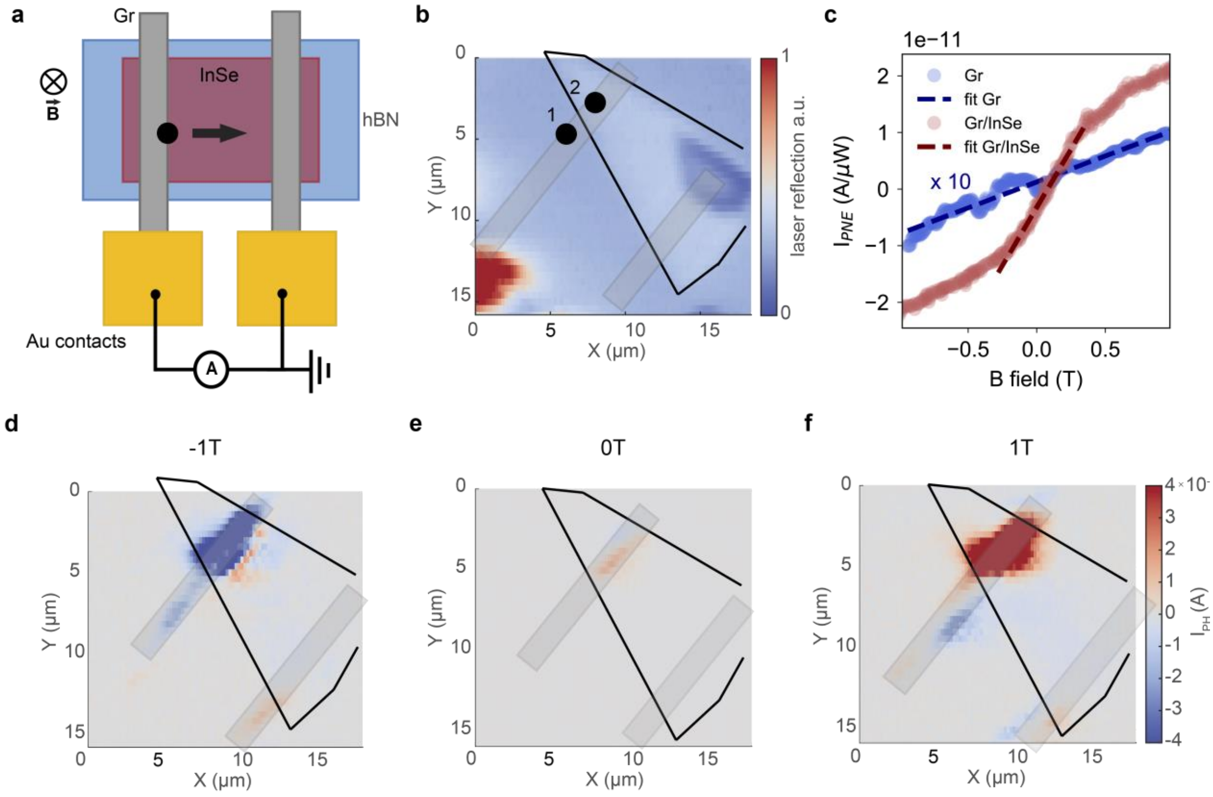

Figure S4. Scanning photocurrent map of the Nernst effect across an InSe channel: (a) Device schematic showing the illumination of the Gr/5L-InSe heterostructure and electrical detection across the InSe channel. Following this schematic, any measured current is forced to flow through the semiconductor. (b) Laser reflectance map of the region of interest measured simultaneously with the scanning photocurrent map. This measurement allows us to correlate the position of the laser with the signal observed. The positions chosen to record the Nernst effect signal on graphene and on the InSe/graphene heterostructure are labeled as position 1 and 2, respectively (c) Nernst effect signal recorded varying magnetic field and under 50µW of laser illumination and V_g=0 V in position 1 and 2, shining light on the graphene electrode and on the heterostructure, respectively. The bare graphene signal is shown in blue, magnified by an order of magnitude to better highlight the difference in slope between the two curves. The measurements are performed without any applied bias as it would obscure the Nernst effect, inducing other photocurrent mechanisms in the picture. (d) A scanning photocurrent map showing the measured photocurrent across the full device at -1T of applied out-of-plane electric field. (e) and (f) analogous scanning photocurrent map shown for 0T and 1T, respectively.

This measurement was attempted also on the 3L InSe device, without success. The reason behind this discrepancy lies in the extremely high conductivity of the 5L device, higher than

the 3L and comparable to graphene while switched on. Another parameter that did not allow the observation of PNE current in the 3L device in the transistor geometry is that the channel, which is more resistive than 5L InSe, is longer, hence further reducing the magnitude of such a signal.

The photovoltage and photocurrent correspondence was further confirmed by measuring the same signal shown in Figure 3 of the main text but in the photovoltage configuration, as shown in Figure S3. Indeed, the two measurements show equivalent results, further confirming our analysis. The correspondence is shown in logarithmic scale since it highlights the hotspots, and all the features are clearly visible. Such a result clearly indicates that analogous information can be obtained from the device while measuring in both geometries.

The photo-Nernst effect in graphene devices enables the generation of a thermoelectric photocurrent through a localized current density source. This effect arises due to the interplay between the electron-phonon scattering and the electron density in graphene. By tuning the Fermi level using a gate voltage, the available states for electron-phonon scattering change, affecting the efficiency of the photo-Nernst effect.

It is worth mentioning that the photo-Nernst effect in graphene is subject to certain limitations. In particular, its effectiveness heavily depends on the presence of inhomogeneities or junctions within the graphene device. These localized current density sources are crucial for driving the photocurrent. For this reason, scanning photocurrent maps of the PNE often show hotspots where the effect is more prominent. In the absence of such inhomogeneities or in pristine, homogeneous graphene, the photo-Nernst effect is considerably diminished or may even be absent. Therefore, the intentional design of specific device architectures or introducing defects in the graphene structure might be necessary to enhance the photo-Nernst effect.

As mentioned, defect states can act as scattering sites that can enhance the photocurrent generation. In InSe, the most common defect type is the Selenium vacancy, as extensively studied in our previous works[3,4]. In particular, three defect states are present within the bandgap, with two levels appearing at energies close to the conduction band, and one level appearing close to the valence band. Indeed, upon saturation of these defects by tuning the Fermi level and applying positive gate values, such scattering sites cannot contribute any more to the signal, providing a possible explanation for the quenching of the PNE in our system as detailed in the main text.

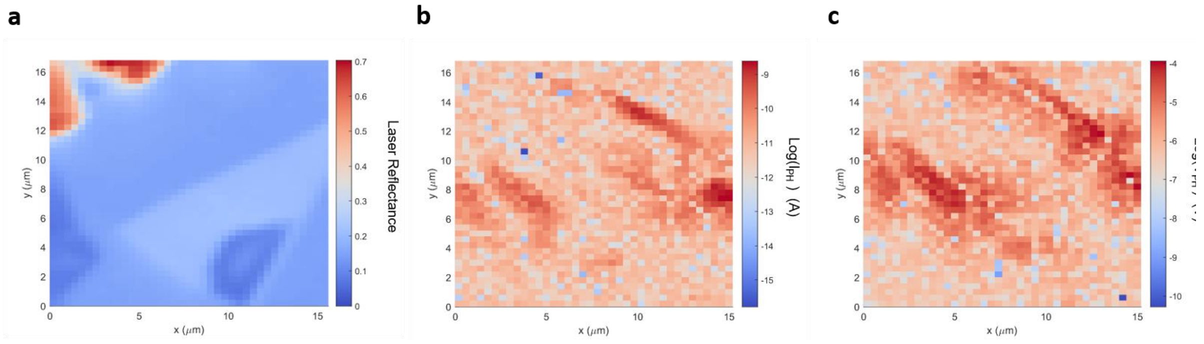

Figure S5. Relation between photovoltage and photocurrent maps. (a) Laser reflectance map of the 5L InSe device from the main text. (b) Scanning photocurrent map of the same device, plotted in logarithmic scale to better highlight all the features. (c) Scanning photovoltage map that provides analogous information with respect to the photocurrent map. The values are plotted in logarithmic scale to compare effectively with the photocurrent map. Both (b) and (c) are measured at 100mK and 1T.

Another aspect to consider is the assumption of rapid electron thermalization inherent in the formalism of the photo-Nernst effect. While this assumption is valid for graphene and InSe due to their unique electronic properties, it may not hold true for other materials. Therefore, careful

consideration should be given to the applicability of the photo-Nernst effect formalism when applying it to different systems.

Despite these limitations, the photo-Nernst effect in graphene holds significant potential for various applications. The ability to generate a photocurrent in graphene, a gapless material, opens up new avenues for optoelectronic devices and energy harvesting systems. Leveraging the tunable photo-Nernst effect, it becomes feasible to develop graphene-based photodetectors, sensors, and energy conversion devices that operate efficiently in the terahertz and infrared frequency ranges. Further research and development endeavors are warranted to fully explore and exploit the practical applications of the photo-Nernst effect.

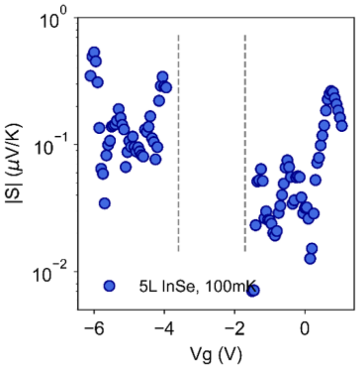

Figure S6. Seebeck coefficient of 5L InSe. (a) Seebeck coefficient of 5L InSe calculated with respect to Gr. The values are reported in module, in order to show both the electron (S < 0) and hole sides (S > 0).

3. Thermoelectric signal: Seebeck and Nernst effect calculations

In order to evaluate the Nernst and Seebeck coefficients in our system we borrow the physics employed for semiconductors and graphene. So far, the two systems have been treated separately, but we can take advantage of the Gr/InSe to model the temperature gradient at the junction. The difference in T obtained at the junction is the heat generated by the laser, analogously to the case of MoS_2 as previously observed[5]. However, such a temperature gradient is also the same one responsible for generating the photo-Nernst effect, thus allowing us to estimate the Nernst coefficient with equations S3 and S4.

The first step is the calculation of the Seebeck coefficient, starting from the conductance variation as a function of gate voltage, following the Mott approximation. As briefly mentioned in the main text, the Mott relation doesn't produce a perfect agreement with the experimental values obtained previously in InSe[6]. However, the calculated values show a discrepancy of a factor of 2 in the worst case, with reasonable agreement when the carrier density is around $1.5 \cdot 10^{12}\ cm^{-2}$, which is a value easily accessible in our experiments. In our case, this calculation would yield a value larger than the real Seebeck coefficient, which in turn, would give a higher ΔT with respect to our case, and hence decrease the value of the calculated Nernst coefficient by a maximum of 1/2. Moreover, we use the calculated value to compute the Nernst coefficient, and we use a factor β that dampens the total obtained value, thus yielding a lower bound for the Nernst coefficient value. A discrepancy of a factor of 2 is thus compensated by the β factor, which we set to 0.5β to obtain the numerical results keeping into account such a discrepancy. Furthermore, the Mott formula is known to describe more accurately systems at low temperatures with respect to room temperatures, since scattering mechanisms due to lattice vibrations are reduced. Hence, we justify the employment of the Mott formalism since it doesn't affect the main message of our work, namely a tunable Nernst effect device operating at millikelvin temperatures.

Keeping into account the motivations above, we write the Seebeck coefficient as follows:

$$S_{InSe} = \frac{\pi^2 k_b^2 T}{3e} \frac{1}{G} \frac{dG}{dV_g} \frac{dV_g}{dE_F} \quad \text{(S5)}$$

For the 3L InSe, the results are presented in Figure 2c of the main text. For the 5L InSe, the results are shown in Figure S6 for 100 mK. We note that when the device is in the off state, the resistance of the device is comparable to the input impedance of the instrument. Thus, the values obtained there are not reliable and are not shown.

In particular, since InSe has an asymmetric band shape between the electron bands and hole bands, care must be taken in the factor $\frac{dV_g}{dE_F}$. Since the hole side factor is much larger than that of the electron side, we split the two contributions on opposite sides of the off-state and rescale based on the band topology.

$$S_{InSe\_n} = \frac{\pi^2 k_b^2 T}{3e} \frac{1}{G} \frac{dG}{dV_g} \left. \frac{dV_g}{dE_F} \right|_n \quad \text{(S6)}$$

$$S_{InSe\_p} = \frac{\pi^2 k_b^2 T}{3e} \frac{1}{G} \frac{dG}{dV_g} \left. \frac{dV_g}{dE_F} \right|_p \quad \text{(S7)}$$

The next step consists of measuring the I_s-V_s curves at $V_g = 0$ with laser illumination on, and laser wavelength below the bandgap, to ensure that only a thermal signal is present. Indeed, we observe a signal only where the electrodes are present, with a change in sign typical of the thermoelectric signal. Below, two representative scanning photocurrent maps are shown for the 3L and 5L devices. The signals are recorded simultaneously with the laser reflectance image, in order to compare and attribute the spatial position to each point. The bandgaps of 3L and 5L InSe are 1.67eV and 1.48eV, respectively. Hence, laser wavelengths below those energies are employed to avoid absorption in the semiconductor.

The CW laser wavelength used is 904 nm for both the 5L InSe and 3L InSe, and a further check has been performed with an 810 nm laser for the 3L, and the laser power is kept to the same value used for the PNE measurements, namely 50 µW. The same signal is further measured at 100 µW, to compare the reliability of the measured signal. We note that, while changing laser wavelength, it is crucial to make sure that the power meter detecting the power has a flat response over the desired wavelength range, to avoid a power mismatch between the irradiating power and the detected one. The photocurrent/photovoltage measured in the scanning photocurrent maps can be compared with the previously measured I_s-V_s curves, measured under the illumination of the 904nm laser, shown in Figure S8. We note that the error obtained from the fit will be propagated to the thermal gradient and thus to the Nernst coefficient.

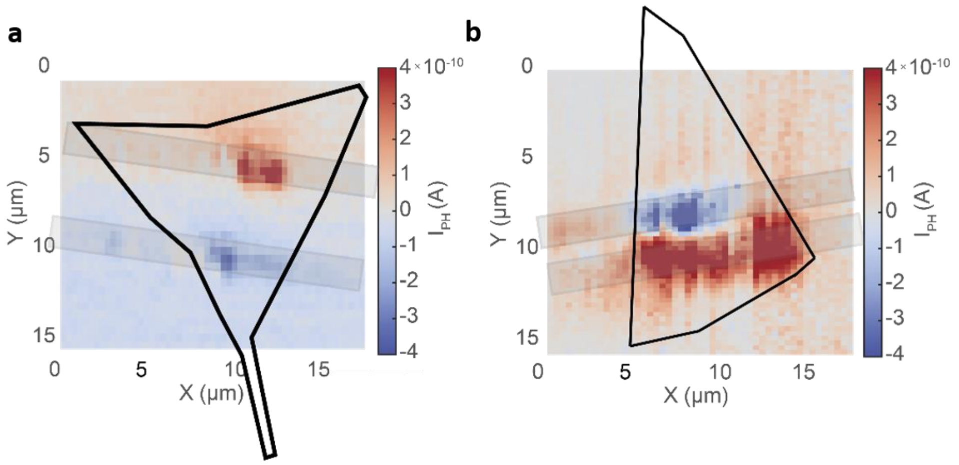

Figure S7. Scanning photocurrent map at energies below the bandgap of InSe. (a) Scanning photocurrent map measured with laser excitation energy below the bandgap energy for the 3L InSe device. Laser wavelength 904 nm. (b) Signal analogous to (a), measured for the 5L InSe device at a laser wavelength of 904nm.

From the spatial maps (Figure S7), and the I_s-V_s curves (S8), we can extract the corresponding bias voltage values that were produced by the laser light at any point. Then, we can compute the temperature difference for each point using:

$$V_{th} = \Delta T \cdot (S_{InSe} - S_{Gr}) \tag{S8}$$

The resulting line cuts and thermally generated voltages are shown in Figure S9.

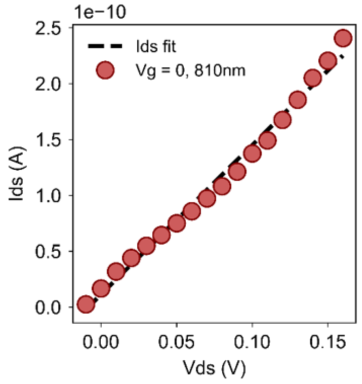

Figure S8. Effective bias potential extraction. (a) Drain-Source current as a function of the drain-source bias recorded while shining the 810nm laser onto the channel. The plot is used to compare the thermoelectric signal to the effective bias generated at the junction by the laser during the photo-Nernst measurements.

The temperature difference that generates the thermal gradient is therefore known, which serves as the driving force for the photo-Nernst Effect. We note that since the calculated Seebeck effect is an upper bound of the real value, the thermal gradient we calculate represents a lower bound value of the ΔT, and thus the real thermal gradient is likely larger, further showing an enhancement of the Nernst coefficient in our device. Knowing ΔT, B, l, and w, we can compute the values of βN for our devices:

$$\beta N \propto I_{ph} \cdot \frac{\rho_{xx}}{B \cdot \Delta T} \tag{S9}$$

$$\beta N \propto V_{ph} \cdot \frac{w}{l \cdot B \cdot \Delta T} \tag{S10}$$

It is important to stress that all the values computed in this work represent an underestimation of the real Nernst coefficient due to the β factor. Indeed, further studies are required to understand the full potential of such a Gr/InSe heterostructure. From a broader perspective, the results presented in this work can be compared with previous reports of thermopower measurements in different systems, such as spintronic devices. In particular, Tu et al. report values of Seebeck thermopowers of 390 μV/K at room temperature in the antiferromagnetic metal IrMn[7], whereas Sun, Z. et al. show a maximum Seebeck thermopower of around 300 μV/K at 70K, and rapidly decreases when below the Neel temperature[8]. In the case of graphene, the thermoelectric power is also high at room temperature, with values around 300 μV/K, and decreases to 10 μV/K at 40K, reaching values in the order of 1 μV/K around 15K[9]. Moreover, the microscopic reason behind the enhancement observed in this work is not yet well understood, motivating theoretical efforts toward this end.

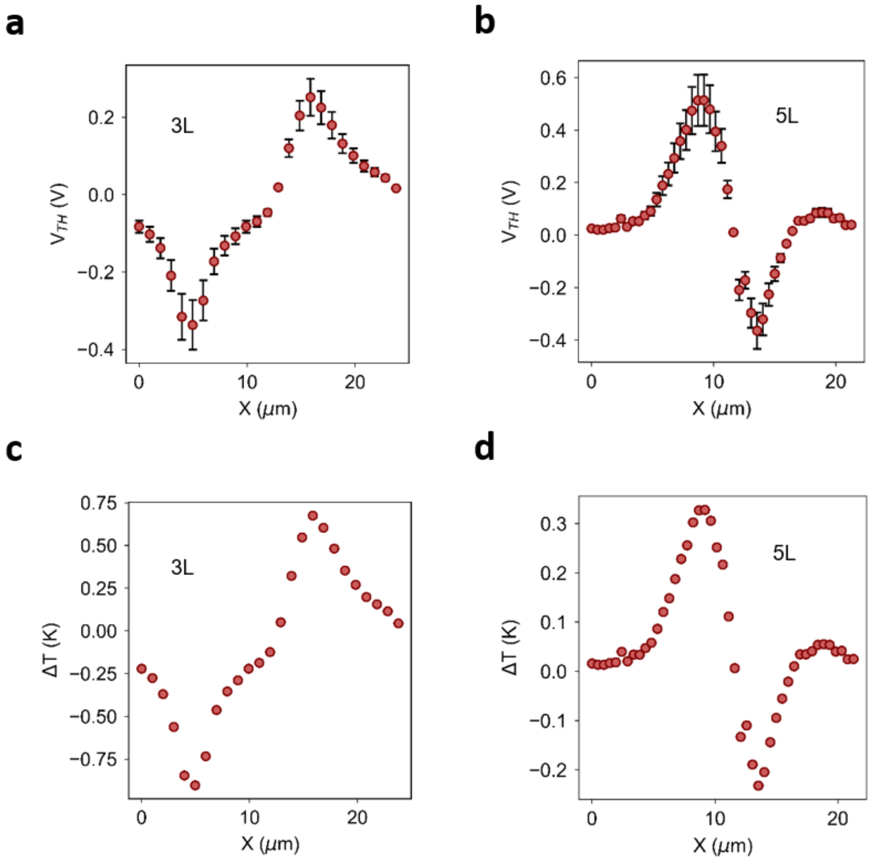

Figure S9. Thermoelectric voltage and temperature gradient. (a) Line cut of the extracted thermoelectric voltage for the region of interest in the 3L device. (b) Signal analogous to (a), measured for the 5L InSe device. Laser wavelength 904nm. The error bar represent the error in the estimation of the thermal gradient, which is propagated to the thermoelectric voltage. (c) Estimated thermal gradient as calculated with equation S8 for 3L InSe. (d) Estimated thermal gradient for 5L InSe.

4. Seebeck and Nernst Power factor

The Seebeck and Nernst power factors, as described in previous reports[10], are defined as:

$$PF_S = S_{xx}^2 \sigma_{xx} \tag{S11}$$

$$PF_N = S_{xy}^2 \sigma_{xy} \tag{S12}$$

Where the S_{xx}^2 refers to the Seebeck coefficient, $S_{xy}^2 = N^2$ is the Nernst coefficient, and σ_{xx}, σ_{xy} are the electrical conductivities of the longitudinal and transversal channel, respectively.

For the 3L the Seebeck power factors are computed at 300 K and 100 mK and the results are shown in Figure S10. Notably, the PF values are also very high, comparable to the best reported in the literature.

Furthermore, the figure of merit zT of our devices is computed using a thermal conductivity of InSe of 10 $W\ m^{-1}K^{-2}$, which is a reasonable value for in-plane thermal conductivity of InSe as reported in the literature[6,11,12]. The results are also shown in Figure S10.

For the Nernst Power Factor, the 5L InSe/Gr can be evaluated only at V_g = 0 V. At that gate value, the result obtained by Equation S10 yields $PF_{N,5L} = 2.08\ \mu W\ m^{-1}K^{-2}$. For the 3L InSe/Gr heterostructure, we can evaluate the PF_N while changing the carrier density. In Figure S11 we show the PF_N versus gate voltage and versus conductivity for the p-type conduction. In particular, we show that the p-type values increase dramatically when entering in the hole conduction state, with an exponential onset of the power factor. We further plot the PF_N as a function of conductivity in logarithmic scale, analogously to the Seebeck counterpart. A linear trend is observed, which is consistent with previous reports of PF_N.

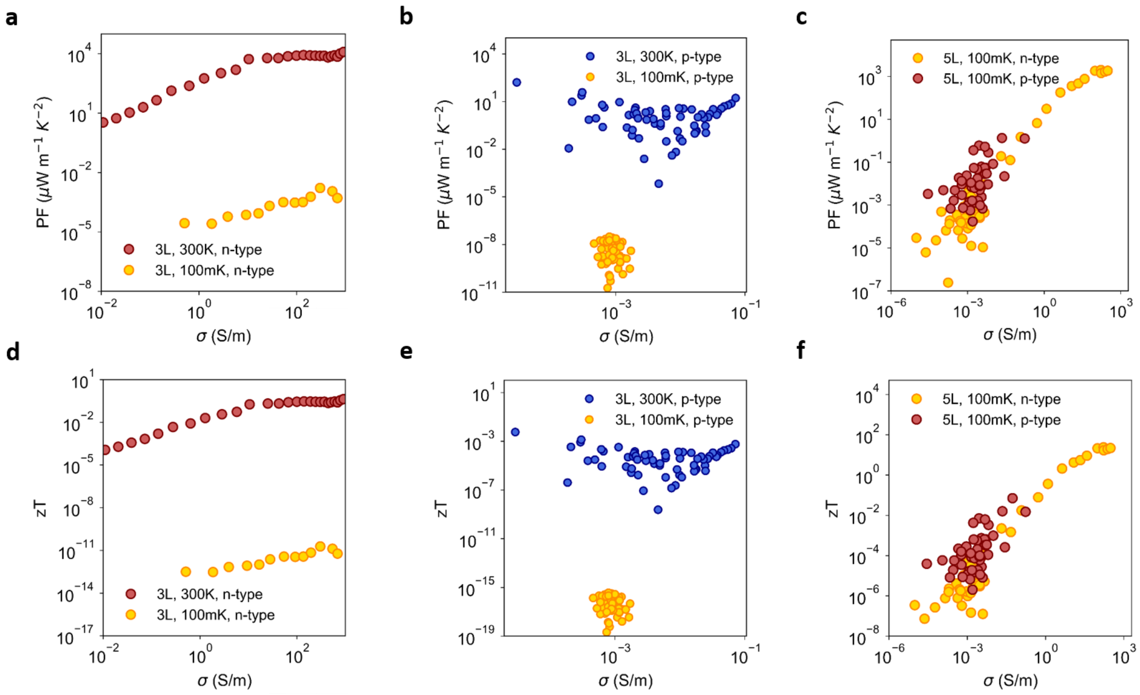

Figure S10. Power factor and figure of merit of InSe devices. Power Factor of the Seebeck coefficient of 3L (a) and (b) and 5L (c) InSe. Both electron and hole sides are shown. (d) Figure of merit zT calculated for the n-side of 3L InSe at 100mK and 300K. (e) Analogous to (d) but for the p-side. (f) Figure of merit of 5L InSe at 100mK, both electron and hole sides are shown.

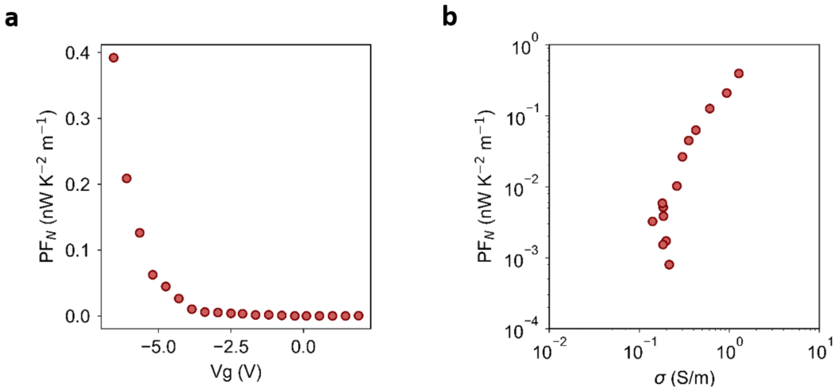

Figure S11. Nernst Power factor of InSe devices. (a) Nernst Power Factor of 3L InSe/Gr as a function of gate voltage. (b) Nernst power factor shown with respect to conductivity.

5. Relation between Fermi energy change and carrier density in InSe

In order to estimate the Seebeck coefficient in our system, the inverse of the factor dE_F/dV_g is required. The computation of such factor was performed in our previous work[3] in the context of photoluminescence spectroscopy measurements. First, we quantify the redshift observed in the p-doped regime as a function of the Fermi energy E_F. Based on the thickness (t) and dielectric constant (ε_{hBN}) of hBN, we can estimate the geometrical capacitance per unit area: $C_g = \epsilon_0\epsilon_{hBN}/t = e\, dn_h/dV_g = 0.0012\, F/m^2$, where n_h is the carrier density of holes. The density of states (DOS) in the valence band dn_h/dE_F can be expressed using $dn_h/dE_F = m_h/\pi\hbar^2 = 4.2 \times 10^{14}$ cm^{-2}eV^{-1}. The DOS in the conduction band can be thus estimated by: $dn_e/dE_F = m_e/\pi\hbar^2 = 5.8 \times 10^{13}$ cm^{-2}eV^{-1}. Combining the two equations, we obtain $dE_F/dV_{gh} = 0.0018\, e$, and $dE_F/dV_{ge} = 0.0128\, e$ for 5L InSe. The 3L InSe has slightly higher hole and electron effective masses, but the overall result remains approximately the same.

6. Experimental Setup

The experimental setup used throughout this work is shown in Figure S12 and consists of a dilution refrigerator equipped with a window that allows the laser light to enter the sample space through a set of mirrors. The sample area can be scanned by means of a piezo stage, which allows to perform scanning photocurrent measurements. The incoming laser source is modulated by a chopper, which cuts the beam with a frequency set by the controller. The same chopper frequency is used to drive the lock-in detection of the AC signal generated by the laser.

The sample is connected electrically to a breakout box, which transfers the electrical signal to a Keithley for DC measurements, and to a Lock-in amplifier for AC detection. The reflected light is further collected and sent to a photodiode, in order to measure simultaneously the laser reflectance of the area of interest. Such a simultaneous measurement allows us to correlate the observed scanning photocurrent map with the sample morphology.

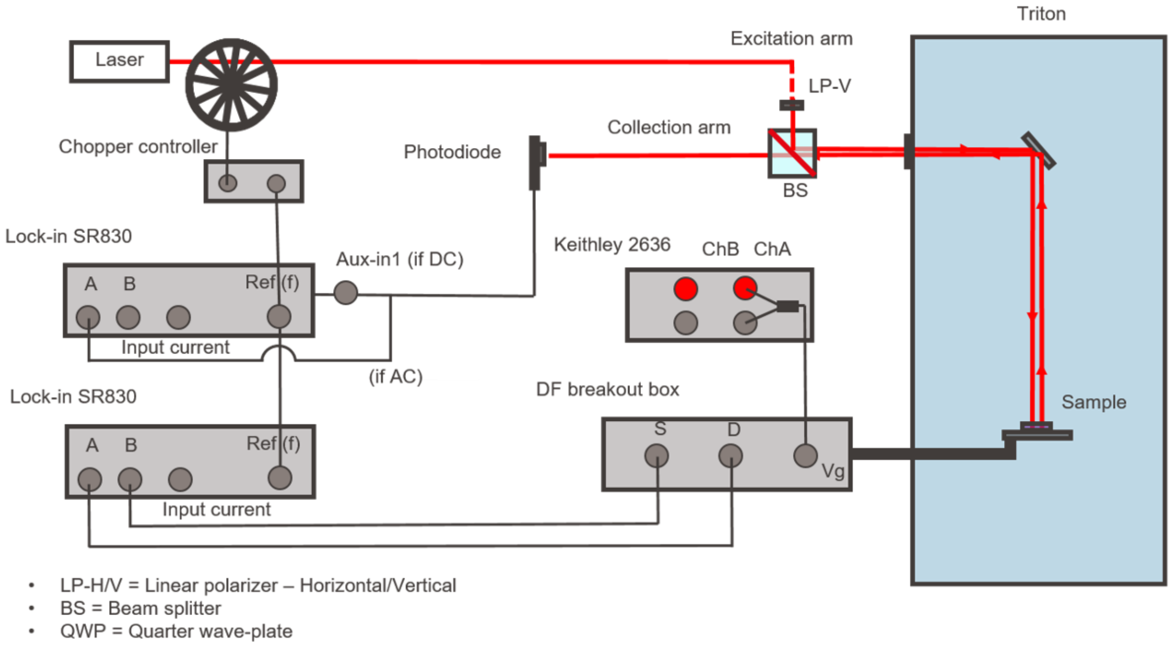

Figure S12. Setup schematic. Schematic of the experimental setup employed in this work.

7. High-Field PNE

The Photo-Nernst measurements required an extensive sweeping of the magnetic field, which was kept between -1 and 1 T to optimize the measurement time. Although not necessary for this work, we attempted high-field measurements to gain a further understanding of the system under study. The measurement requires a very slow magnetic field sweeping, to avoid overheating of the superconducting magnet. Thus, attempting such measurements becomes less reliable the longer they run since the sample vibration and setup instability might cause spurious signals to appear. For this reason, we limited ourselves to reasonably low fields for the majority of the measurements. The higher field sweep from -3 T to 3 T is shown in Figure S13. The signal follows a similar trend with respect to the linear slope observed at a low field. Above |2| T, the PNE signal starts to deviate from linearity, hinting at the start of possible oscillations, which were previously observed in bare graphene[10,13].

The signal is observed on the InSe/Gr heterostructure region, while the gate voltage is kept at -6 V, where the signal is more pronounced.

The signal is observed on the InSe/Gr heterostructure region, while the gate voltage is kept at -6V, where the signal is more pronounced.

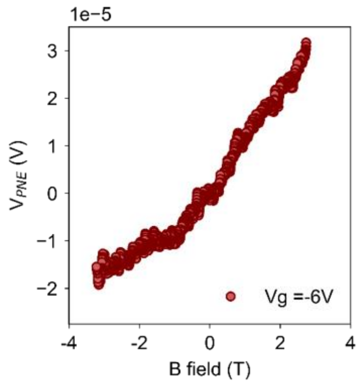

Figure S13. High-field Nernst effect in InSe devices. (a) Photo-Nernst voltage measured at higher B field for incident laser power 150µW. The signal deviates from linearity, hinting at possible oscillations due to the nature of the effect on graphene.

8. Complementary measurements

For completeness, we report the power dependent PNE Intensity as recorded on the Gr/InSe heterostructure, showing a linear trend analogously to the one observed on bare graphene. This observation motivates our approach following the Schokley-Ramo formalism.

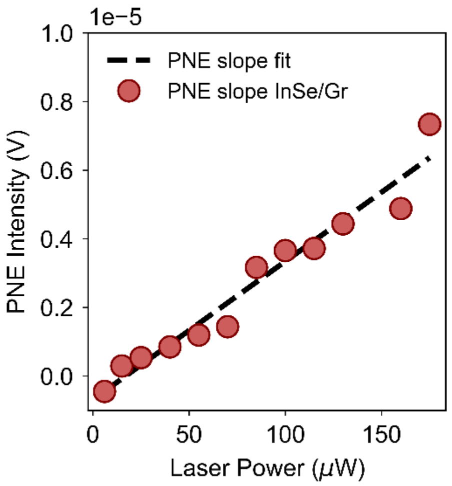

Figure S14. PNE Intensity as a function of laser power recorded on the Gr/InSe heterostructure. The signal is linear in power, analogously to the one recorded on bare graphene as shown in the main text in the inset of Figure 1d, confirming our analysis.

Through this expanded analysis, we aim to provide a more comprehensive understanding of the semiconductor properties of InSe and the coupling effects at play within the graphene/InSe heterostructure, thereby reinforcing the validity of our methodological approach.

We set a gate voltage value and we record the photovoltage while sweeping the magnetic field between -1T and 1T. First, we make sure that no hysteresis is present, and once that is established, we repeat this measurement for multiple gate voltage values. The laser power is kept at 50 µW for the whole experiment. This way, we probe the Nernst effect at fixed carrier densities, and, to the best of our knowledge, the formalism discussed above can be applied to our graphene channel proximitized by InSe. The set of data we record consists of linear plots of the Nernst effect plotted against B as shown in Figure 1d.

In order to obtain a meaningful result, we calculate the slope of these plots, and we show the obtained slopes for different gate voltages, and the result is shown in Figure S15.

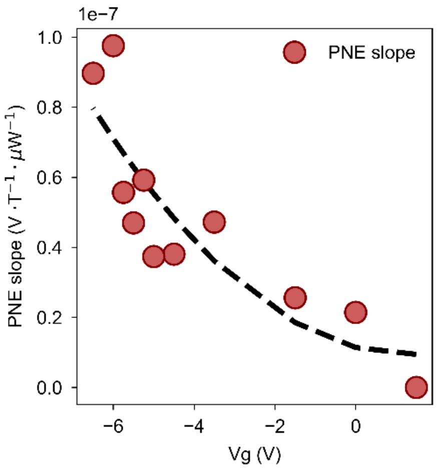

Figure S15. PNE intensity at different gate voltages. (a) The PNE slope is obtained from fitting the magnetic field dependence of the PNE voltage when sweeping the magnetic field from -1T to 1T. The experimental conditions are analogous to those shown in the main text for Figure 2a.

We note that the magnetic field scanning needs to be carefully controlled at a low speed to avoid the overheating of the magnet. Moreover, although our system is particularly stable for sample vibrations, measuring continuously for several hours can yield more inaccuracies with respect to the measurement shown in Figure 2a. Thus, the purpose of this measurement is to show the same qualitative trend with respect to Figure 2a, although with a lower resolution. The observed trend shows a higher signal at gate voltages corresponding to the bandgap and hole conduction of InSe, and a quenched response for voltages corresponding to the electron transport side, further confirming our interpretation. We note that the error bar of the slopes corresponding to each point of the curve in the plot is not shown since it would be misleading as briefly explained below.

The linear signal dependence in the B field, as shown in Figure 1d, is characterized by a limited error bar as shown here below in Figure S16.

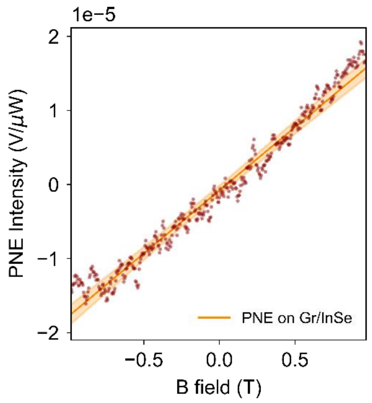

Figure S16. PNE intensity as a function of magnetic field. The PNE intensity is shown together with the fit while varying the magnetic field from -1T to 1T. The experimental conditions are analogous to those shown in the main text for Figure 1d. The error bar of the fit cannot describe the uncertainty observed in Figure S14 as discussed in the explanation below.

However, over long measurement times in the order of a day, the inaccuracies due to challenging experimental conditions overshadow the error of the fit. For this reason, the change in slope can be considered to be qualitatively meaningful but should not be considered to quantitatively describe our system. For this reason, an analogous and more reliable measurement is shown in the main text (Figure 2a), where the measurement time is contained since the carrier density is changed at a fixed magnetic field.

In addressing the observations related to the second geometry illustrated in Figure 3a, we recognize that the behavior of the signal, as detailed in Figure 3c and measured at a constant gate voltage, initially suggests adherence to the formalism previously applied. Nonetheless, the emergence of non-linearities at elevated magnetic field strengths signals a deviation when InSe assumes the role of the channel material. This departure from expected behavior emphasizes the limitations of directly applying the established formalism in its entirety under these conditions. However, by confining our analysis to a narrow magnetic field range around B = 0, we observe that the signal retains characteristics akin to those measured on pristine graphene. This includes the antisymmetric dependency on the magnetic field, a hallmark of the photo-induced Nernst effect (and excluding other mechanisms such as the photo-induced Seebeck effect which would be symmetric), thereby lending preliminary support for the applicability of the formalism within this constrained context. For this reason, we compare the slope of the PNE intensity when restricting the magnetic field region near zero, while we show the complete set of data for the sake of completeness and transparency. Our purpose with the employed formalism is to attempt to quantify the effect with respect to the one observed on the bare graphene, which is meaningful in the low-field regime.

To the best of our knowledge, there is no extended formalism that would allow us to describe this system, and we thus encourage theoretical efforts toward this end. The theoretical description of such a system would require both theoretical and computational models that are out of the scope of this work, due to the complexity of the formalism involved.

9. Possible origin of the enhancement and influence of the van Hove singularity

From previous theoretical studies, it is known that the van Hove singularity is linked to enhanced thermoelectric performances, such as an increase in the Seebeck coefficient[14]**.** An increased Seebeck coefficient in InSe would cause a higher disparity between the Seebeck coefficients of graphene and InSe itself. Thus, we expect an additional thermoelectric voltage to appear at the junction, which is dependent on the charge configuration of InSe and enhanced by the singularity.

The enhancement of thermoelectric properties in few-layer indium selenide (InSe) near the van Hove singularity at the valence band edge can be qualitatively understood through mechanisms similar to those observed in twisted bilayer graphene (TBG) but with distinct material-specific characteristics. In few-layer InSe, the presence of a van Hove singularity at the valence band edge introduces sharp features in the electronic density of states (DOS), similar to the flat bands near the magic angle in TBG. These sharp DOS features significantly influence transport properties by leading to a narrow transport distribution function (TDF), where states are concentrated around the van Hove singularity, thereby improving the Seebeck coefficient and maximizing the power factor[15].

The relationship between the enhancement of thermoelectric properties and the electronic structure in few-layer InSe can also be described by considering the trade-off between the increased DOS at the van Hove singularity and the carrier mobility. While the high DOS at the singularity boosts the Seebeck coefficient and electrical conductivity, the mobility of carriers is crucial for maintaining high thermoelectric efficiency. In few-layer InSe, the carrier mobility might be affected by the proximity to the van Hove singularity, requiring a balance to optimize the thermoelectric performance. We can implement this reasoning to show that, from the theoretical interpretation hereby presented, an enhancement of the Seebeck coefficient is expected.

From the Mott relations discussed in Supplementary Equation S5, the Seebeck coefficient is defined as $S_{InSe} = \frac{\pi^2 k_b^2 T}{3e} \frac{1}{G} \frac{dG}{dV_g} \frac{dV_g}{dE_F}$, which can be rewritten as $S_{InSe} = \frac{\pi^2 k_b^2 T}{3e} \frac{\partial ln\sigma(E)}{\partial E}\Big|_{E_F}$.

Now we consider the conductivity, which has the form:

$$\sigma(E) = \frac{1}{\Omega(2\pi)^2 F} \int v(k)g(k)\delta\big(E - E(k)\big)d^2k \tag{S13}$$

Where Ω is the volume of the first Brillouin zone, is the applied electric field, $v(k)$ represents the group velocity of carriers (electrons or holes) at a given wave vector k in the material's band structure, $g(k)$ is the distribution function of carriers in momentum space. It describes the probability of finding a carrier with a specific momentum k, and $\delta(E - E(k))$ is the Dirac delta function, which is zero everywhere except at $E = E(k)$, where it is infinite but integrated to unity. In this context, $\delta(E - E(k))$ ensures that the integral in the equation selects only the contribution from states with energy $E(k)$ that match the desired energy[15]. Following the results by Hwang et al.[16], near the Fermi level, the conductivity can be approximated as:

$$\sigma(E_F) \approx \left(\frac{e}{2}\right) v_F(E_F)D(E_F)/S_0 \tag{S14}$$

where S_0 is the total scattering rate (inverse of relaxation time) and $D(E_F)$ is the density of states.

Thus, the Seebeck coefficient is $S \propto D'\ (E_F)/D(E_F)$. This proportionality implies that the Seebeck coefficient will be more pronounced when the Fermi level is near a sharp discontinuity in the DOS, in this case, the van Hove singularity at the valence band edge of InSe. We note that since this formalism is valid for graphene (and degenerate metals), it reflects our first device geometry where the graphene is probed (Figure 1a).

However, we propose a tentative extension of this formalism to our second device geometry, premised on treating InSe as a metal-like conductor at the specific gate voltage applied in our experiments. This approximation is deemed reasonable within the context of our study, a perspective supported by the observed resistance of approximately $\sim 30\ k\Omega$, as detailed in Supplementary Figure 3.

The physics we have outlined, although just to a first approximation, gives a clear idea of the influence of the van Hove singularity in our system. Despite this reasoning, obtaining a quantitative agreement that would allow us to claim such an origin of the enhancement is not within our reach so far. The precise characterization of the underlying mechanisms contributing to this phenomenon necessitates further investigation.

10. Ultra-low temperature applications

In the context of qubit circuits, one of the main engineering challenges is that the qubits require an ambient temperature of around ~100 mK, while the Cryo-CMOS technology needed to electrically control the qubits requires cooling powers that are not available at such low temperatures[17]. Thus, the control circuitry is operated at higher temperatures (4K), where more efficient cooling powers are currently available[18,19]. This architecture requires numerous efforts to interface the two environments and to ensure proper functioning and lossless transmission of the signals of interest. Ideally, the best case would be to operate the micrometer-sized electronic circuitry at 100mK together with the qubits. To this end, more efficient thermoelectric materials and devices are needed with respect to the currently available ones, to allow the implementation of such an architecture.

Our device schematic and materials, as presented in Figure 1a, suggest that graphene/InSe heterostructures can be used to convert localized heat sources into electrical signals at ultra-low temperatures, which would be suitable for the possible operation of the Cryo-CMOS circuitry in such an environment. Moreover, if designed properly, our device could convert

electrical signals into controlled temperature gradients that could be engineered to effectively cool down the components of interest at ultra-low temperatures. The conversion process can be summarized as follows. In our experiments, the heat is generated by the laser spot, which produces a radial heating with a zero net directional gradient, unless the laser is shone on the edge of the material. In this case, an effective thermal gradient is present, driving the Nernst effect. In a real quantum circuit, our devices could be implemented e.g. positioned below the heat hotspots of the circuit, with the flake edges carefully positioned below the heat generating point. In this case, the heat generated in the circuit serves as an out-of-plane generating field for driving the Nernst effect, which allows the generation of an electrical current/voltage that can be employed either as a sensor or as a further component of the circuit.

We note that the definition of the efficiency of such a process depends on the system under study, and thus requires careful examination of the given circuit. However, a general guideline can be defined based on the geometry involved. If the heat gradient is orthogonal to the generated electric field (electrical current) and orthogonal to the applied magnetic field, the Nernst coefficient defines the efficiency of the process. On the other hand, if the heat gradient is parallel to the charge flow, the Seebeck formalism can be employed. The Power Factor can further be employed to compare the efficiencies of the effect, as shown in Supplementary Note 4. If the thermal conductivity is known, the figure of merit (ZT) is also a good estimation of the effect. We suggest that, in general, the power irradiated on the device, divided by the generating fields is a good metric to assess the performance of the device.

11. Simulation of heat transport in InSe/Graphene heterostructure

To model the laser-induced heating in InSe/graphene heterostructures, we initially simplify the simulation by separating the optical and heat transport phenomena. To achieve this, we incorporate absorption coefficients ($\alpha_{graphene}$ and α_{InSe}) to quantify the conversion of absorbed optical energy into heat within each layer. Assuming that each layer absorbs a fraction of the optical power (P_{λ}) and transmits the remainder (1 - α), we develop the model depicted in Figure S17, illustrating the light's trajectory and the energy transmitted through the layers, including direct transmission and reflection on the silicon substrate. While a more detailed analysis would involve considering back reflections and resultant interferences at each interface, we opt to disregard this effect due to its minimal impact on the overall energy calculation.

Therefore, regarding the previous considerations, the total absorbed energy in the InSe, top and bottom graphene can be written as:

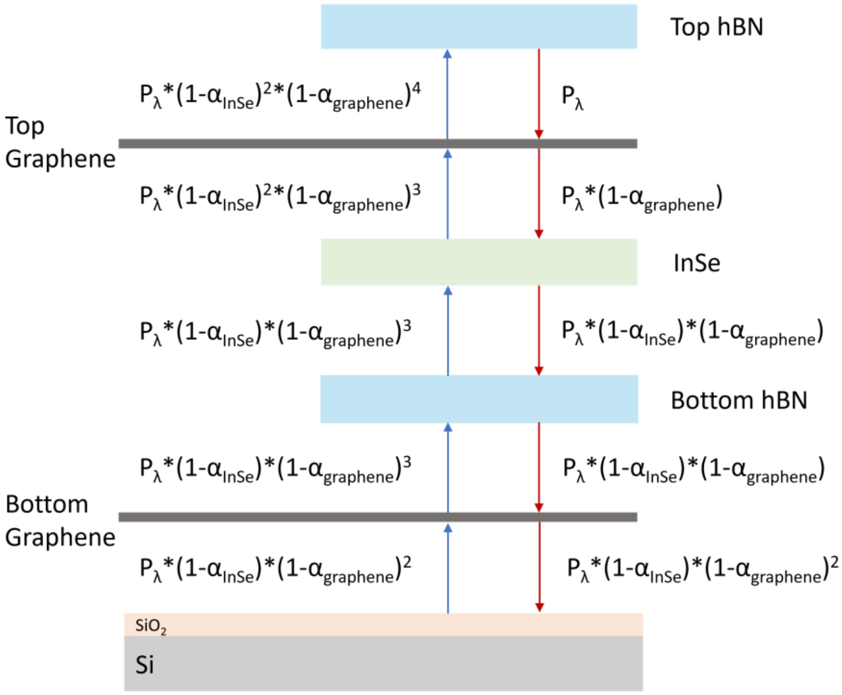

Figure S17. Schematic of the modelled power transfer. Modelling of the optical energy transmission on the InSe/graphene heterostructure.

Top Graphene:

$$P_{ab}^{(top\ graphene)} = P_\lambda \cdot \left[\alpha_{graphene} \cdot (1 - \alpha_{InSe})^2 \cdot \left(1 - \alpha_{graphene}\right)^3 + \alpha_{graphene}\right]$$

InSe:

$$P_{ab}^{(InSe)} = P_\lambda \cdot \left[\alpha_{InSe} \cdot \left(1 - \alpha_{graphene}\right) + \alpha_{InSe} \cdot (1 - \alpha_{InSe})^2 \cdot \left(1 - \alpha_{graphene}\right)^3\right]$$

Bottom Graphene:

$$P_{ab}^{(bottom\ graphene)} = P_\lambda \cdot \left[\alpha_{graphene} \cdot \left(1 - \alpha_{graphene}\right) \cdot \left(1 - \alpha_{InSe}\right) + \alpha_{graphene} \cdot (1 - \alpha_{InSe}) \cdot \left(1 - \alpha_{graphene}\right)^2\right]$$

Subsequently, we employ COMSOL to compute the heat transport within the heterostructure subjected to laser heating. Utilizing the dimensions outlined in Table 1 and the physical properties detailed in Table 3, we construct a model of the heterostructure. Figure S17 visually represents the simulated heterostructure.

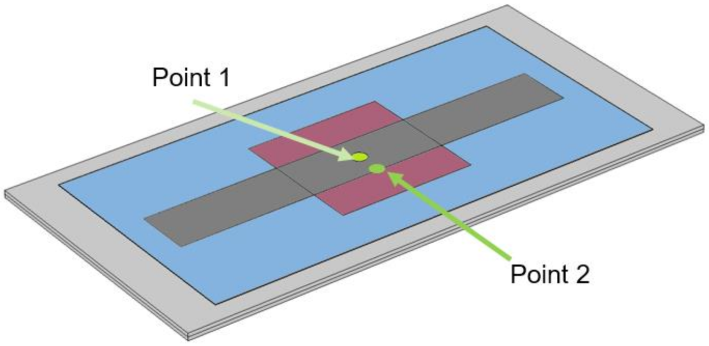

Figure S18. COMSOL rendering of the modelled system. The COMSOL 3D rendering of the heterostructure shows the geometry we employed for the simulations.

Device part	Width	Length	thickness
Top hBN	17 µm	34 µm	20 µm
Top Graphene	4 µm	30 µm	0.35 nm
InSe	10 µm	10 µm	3.6 nm
Bottom hBN	17 µm	34 µm	20 nm
Bottom Graphene	17 µm	34 µm	0.35 nm
Substrate SiO_2	40 µm	40 µm	270 nm
Substrate Si	40 µm	40 µm	270 nm

Table 1. **Dimensions of the heterostructure components**. The physical dimensions of all the building blocks of the device under study are listed in this table. The dimensions correspond to the 3L device shown in the main text.

Physical Parameters			
**Graphene**	Thermal conductivity (k)	100	W/(K.m)
	Heat Capacity (C_{gr})	710	J/(kg.K)
	ρ_{gr}	2300	kg/m^3
**InSe**	Thermal conductivity (k)	8.5	W/(K.m)
	Heat Capacity (C_{InSe})	800	J/(kg.K)
	ρ_{InSe}	5600	kg/m^3
**hBN**	Thermal conductivity (k)	0.2	W/(K.m)
	Heat Capacity (C_{hBN})	700	J/(kg.K)
	ρ_{hBN}	2180	kg/m^3

Table 2. Thermoelectric properties of the materials. The table shows the main thermoelectric quantities of the materials used in the simulation.

Power			
**Laser**	Power	50	μW
	Spot size	1	μm^2
**Absorption**	$\alpha_{graphene}$	0.02	
	α_{InSe}	0 – 0.1	

Table 3. Laser and absorption information. The table shows the laser properties and the absorption characteristics considered in this work. The InSe absorption is varied from 0 to 0.1 to incorporate different scenarios.

The laser is represented as a cylinder with uniform material and thickness within each layer where light is absorbed. Details regarding the laser and absorption coefficients are provided in Table 3. The absorption coefficient of InSe was estimated in our previous work[3], and it is varied from 0 to 0.1 to describe different scenarios. In fact, $\alpha_{InSe} = 0$ reflects the condition when the laser excitation energy is below the bandgap energy of InSe, and thus no absorption is present. The $\alpha_{InSe} = 0.1$ represents the higher bound of the absorption of InSe.

The initial temperature of the system is set at 100 mK, with the back silicon serving as a heat sink, maintained at the same temperature throughout the simulations. Likewise, the edges of the top graphene are fixed at 100 mK to simulate the heat dissipation effect of the drain-source contacts. Given the high aspect ratio of the structure, meshing is conducted on the faces and propagated in the z-direction.

We simulate the conditions that are relevant for the heat dissipation in the first geometry described in the main text, corresponding to the rendering shown in Figure S18. We consider first the scenario where the laser spot is at the center of the graphene strip, as highlighted in point 1 in Figure S18. In these conditions, we simulate the heat transport for different absorption coefficients of InSe, ranging from $\alpha = 0$ to $\alpha = 0.1$, which correspond to the extremes of absorption of 3L InSe at different wavelengths as discussed previously. The result shows a rapid decay of the temperature outside of the laser spot, with temperature differences in the order of 1K, in agreement with our experimental estimate (Figure S9) and supporting our interpretation. Such an in-plane thermal gradient is not compensated when the laser spot is at the edge of the graphene (19b), thus generating the in-plane gradient that contributes to the Nernst effect.

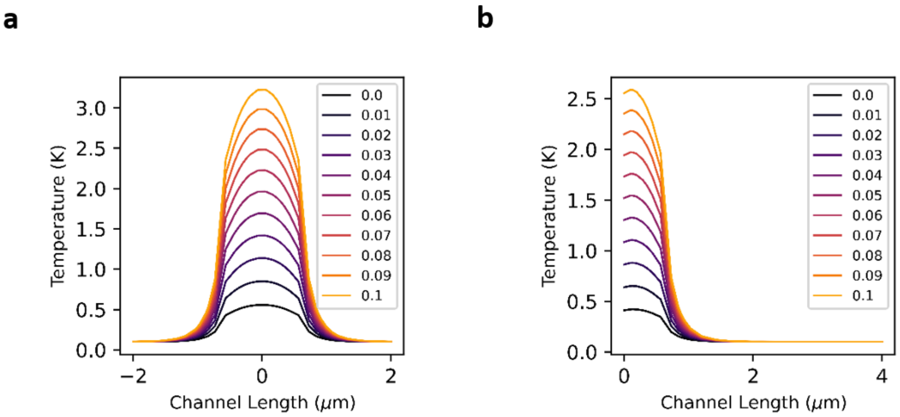

Figure S19. COMSOL simulation of heat propagation. The result of the simulation is presented for the two cases as discussed in the text. (a) Heat propagation and thermal gradient when the laser spot is in the middle of the graphene flake on top of the heterostructure in point 1 as shown in Figure S18. The result of the simulation for the different absorption coefficients of InSe is shown. (b) Analogous study as the one presented in (a) but for point 2 as shown in Figure S18.

12. References

(1) Bandurin, D. A.; Tyurnina, A. V.; Yu, G. L.; Mishchenko, A.; Zólyomi, V.; Morozov, S. V.; Kumar, R. K.; Gorbachev, R. V.; Kudrynskyi, Z. R.; Pezzini, S.; Kovalyuk, Z. D.; Zeitler, U.; Novoselov, K. S.; Patanè, A.; Eaves, L.; Grigorieva, I. V.; Fal'ko, V. I.; Geim, A. K.; Cao, Y. High Electron Mobility, Quantum Hall Effect and Anomalous Optical Response in Atomically Thin InSe. *Nat. Nanotechnol.* **2017**, *12* (3), 223–227. https://doi.org/10.1038/nnano.2016.242.

(2) Cao, H.; Aivazian, G.; Fei, Z.; Ross, J.; Cobden, D. H.; Xu, X. Photo-Nernst Current in Graphene. *Nat. Phys.* **2016**, *12* (3), 236–239. https://doi.org/10.1038/nphys3549.

(3) Pasquale, G.; Sun, Z.; Čerņevičs, K.; Perea-Causin, R.; Tagarelli, F.; Watanabe, K.; Taniguchi, T.; Malic, E.; Yazyev, O. V.; Kis, A. Flat-Band-Induced Many-Body Interactions and Exciton Complexes in a Layered Semiconductor. *Nano Lett.* **2022**, *22* (22), 8883–8891. https://doi.org/10.1021/acs.nanolett.2c02965.

(4) Pasquale, G.; Lopriore, E.; Sun, Z.; Čerņevičs, K.; Tagarelli, F.; Watanabe, K.; Taniguchi, T.; Yazyev, O. V.; Kis, A. Electrical Detection of the Flat-Band Dispersion in van Der Waals Field-Effect Structures. *Nat. Nanotechnol.* **2023**, 1–7. https://doi.org/10.1038/s41565-023-01489-x.

(5) Buscema, M.; Barkelid, M.; Zwiller, V.; van der Zant, H. S. J.; Steele, G. A.; Castellanos-Gomez, A. Large and Tunable Photothermoelectric Effect in Single-Layer MoS_2. *Nano Lett.* **2013**, *13* (2), 358–363. https://doi.org/10.1021/nl303321g.

(6) Zeng, J.; He, X.; Liang, S.-J.; Liu, E.; Sun, Y.; Pan, C.; Wang, Y.; Cao, T.; Liu, X.; Wang, C.; Zhang, L.; Yan, S.; Su, G.; Wang, Z.; Watanabe, K.; Taniguchi, T.; Singh, D. J.; Zhang, L.; Miao, F. Experimental Identification of Critical Condition for Drastically Enhancing Thermoelectric Power Factor of Two-Dimensional Layered Materials. *Nano Lett.* **2018**, *18* (12), 7538–7545. https://doi.org/10.1021/acs.nanolett.8b03026.

(7) Tu, S.; Ziman, T.; Yu, G.; Wan, C.; Hu, J.; Wu, H.; Wang, H.; Liu, M.; Liu, C.; Guo, C.; Zhang, J.; Cabero Z., M. A.; Zhang, Y.; Gao, P.; Liu, S.; Yu, D.; Han, X.; Hallsteinsen, I.; Gilbert, D. A.; Matsuo, M.; Ohnuma, Y.; Wölfle, P.; Wang, K. L.; Ansermet, J.-P.; Maekawa, S.; Yu, H. Record Thermopower Found in an IrMn-Based Spintronic Stack. *Nat. Commun.* **2020**, *11* (1), 2023. https://doi.org/10.1038/s41467-020-15797-6.

(8) Sun, Z.; Wang, H.; Wang, A.; Lei, B.; Zhuo, W.; Yu, F.; Zhou, X.; Ying, J.; Xiang, Z.; Wu, T.; Chen, X. Large Thermopower Enhanced by Spin Entropy in Antiferromagnet $EuMnSb_2$. *Adv. Funct. Mater.* **2022**, *32* (33), 2202188. https://doi.org/10.1002/adfm.202202188.

(9) Zuev, Y. M.; Chang, W.; Kim, P. Thermoelectric and Magnetothermoelectric Transport Measurements of Graphene. *Phys. Rev. Lett.* **2009**, *102* (9), 096807. https://doi.org/10.1103/PhysRevLett.102.096807.

(10) Li, P.; Qiu, P.; Xu, Q.; Luo, J.; Xiong, Y.; Xiao, J.; Aryal, N.; Li, Q.; Chen, L.; Shi, X. Colossal Nernst Power Factor in Topological Semimetal $NbSb_2$. *Nat. Commun.* **2022**, *13* (1), 7612. https://doi.org/10.1038/s41467-022-35289-z.

(11) Rai, A.; Sangwan, V. K.; Gish, J. T.; Hersam, M. C.; Cahill, D. G. Anisotropic Thermal Conductivity of Layered Indium Selenide. *Appl. Phys. Lett.* **2021**, *118* (7), 073101. https://doi.org/10.1063/5.0042091.

(12) Wickramaratne, D.; Zahid, F.; Lake, R. K. Electronic and Thermoelectric Properties of van Der Waals Materials with Ring-Shaped Valence Bands. *J. Appl. Phys.* **2015**, *118* (7), 075101. https://doi.org/10.1063/1.4928559.

(13) Bridgman, P. W. The Connections between the Four Transverse Galvanomagnetic and Thermomagnetic Phenomena. *Phys. Rev.* **1924**, *24* (6), 644–651. https://doi.org/10.1103/PhysRev.24.644.

(14) Cao, T.; Li, Z.; Louie, S. G. Tunable Magnetism and Half-Metallicity in Hole-Doped Monolayer GaSe. *Phys. Rev. Lett.* **2015**, *114* (23), 236602. https://doi.org/10.1103/PhysRevLett.114.236602.

(15) Kommini, A.; Aksamija, Z. Very High Thermoelectric Power Factor near Magic Angle in Twisted Bilayer Graphene. *2D Mater.* **2021**, *8* (4), 045022. https://doi.org/10.1088/2053-1583/ac161d.

(16) Hwang, E. H.; Rossi, E.; Das Sarma, S. Theory of Thermopower in Two-Dimensional Graphene. *Phys. Rev. B* **2009**, *80* (23), 235415. https://doi.org/10.1103/PhysRevB.80.235415.

(17) Sebastiano, F.; Homulle, H.; Patra, B.; Incandela, R.; van Dijk, J.; Song, L.; Babaie, M.; Vladimirescu, A.; Charbon, E. Cryo-CMOS Electronic Control for Scalable Quantum Computing. In *2017 54th ACM/EDAC/IEEE Design Automation Conference (DAC)*; 2017; pp 1–6. https://doi.org/10.1145/3061639.3072948.

(18) Patra, B.; Incandela, R. M.; van Dijk, J. P. G.; Homulle, H. A. R.; Song, L.; Shahmohammadi, M.; Staszewski, R. B.; Vladimirescu, A.; Babaie, M.; Sebastiano, F.; Charbon, E. Cryo-CMOS Circuits and Systems for Quantum Computing Applications. *IEEE J. Solid-State Circuits* **2018**, *53* (1), 309–321. https://doi.org/10.1109/JSSC.2017.2737549.

(19) Veldhorst, M.; Eenink, H. G. J.; Yang, C. H.; Dzurak, A. S. Silicon CMOS Architecture for a Spin-Based Quantum Computer. *Nat. Commun.* **2017**, *8* (1), 1766. https://doi.org/10.1038/s41467-017-01905-6.
